# Nuclear quantum dynamics of boric acid as probed by a thermal-to-epithermal neutron station

**DOI:** 10.1038/s41598-025-29342-2

**Published:** 2025-11-27

**Authors:** Katarzyna Dziedzic-Kocurek, Michał Silarski, Kacper Drużbicki, Patryk Grabowski, Matthew Krzystyniak

**Affiliations:** 1 Department of Experimental Particle Physics and Applications, Faculty of Physics, Astronomy and Applied Computer Science, M. Smoluchowski Institute of Physics, Jagiellonian University, 30-348 Kraków, Poland; 2 Centre of Molecular and Macromolecular Studies, Polish Academy of Sciences, Sienkiewicza, 112, 90-363 Lodz, Poland; 3 STFC Rutherford Appleton Laboratory, ISIS Neutron and Muon Source, Chilton, OX11 0QX UK

**Keywords:** Limits of detection and quantitation for boron, Boron neutron capture therapy, Neutron Compton scattering, Neutron transmission, Total neutron cross section, Chemistry, Materials science, Physics

## Abstract

Boric acid ($\hbox {H}_3$ $\hbox {BO}_3$, BA), due to its neutron absorption and scattering properties, is used in nuclear medicine and nuclear engineering either as an absorber of thermal neutrons or a moderator of epithermal ones. BA was one of the earliest boron carriers historically evaluated for boron neutron capture therapy (BNCT). Recent advances in neutron spectroscopy and imaging, isotope labelling, and *ab initio* simulation have renewed interest in BA as a model system for pilot studies. Also in terms of BNCT treatment, BA emerges again as an efficient boron compound in the case of selected tumour types. Nuclear quantum dynamics and nuclear quantum effects, such as zero-point energy (ZPE), significantly influence the structure and vibrational properties of BA, and consequently, via Doppler-broadening, alter the way it scatters and absorbs neutrons. In this work, we systematically characterise the nuclear dynamics of boric acid in an isotope-resolved manner across a broad energy range using VESUVIO, a thermal-to-epithermal neutron station at the ISIS facility. We validate a series of thermodynamic and neutron observables against comprehensive *ab initio* calculations, providing direct insight into isotope-dependent neutron scattering and absorption in BA. Moreover, we establish limits of detection and quantification of boron and boric acid by concurrently employing neutron Compton scattering, the incident neutron energy-dependent transmission and prompt-gamma activation analysis.

## Introduction

Boron neutron capture therapy (BNCT) is an innovative binary cancer treatment modality based on the neutron capture reaction of boron-10 ($^{10}$B), which possesses a remarkably high thermal neutron capture cross-section^1^. The nuclear reaction yields high-energy alpha particles ($^{4}$He) and lithium-7 ($^{7}$Li) ions^1^:

1 $$\begin{aligned} ^{10}\text {B} + n_{\text {thermal}} \rightarrow \alpha (^{4}\text {He}) + ^{7}\text {Li} + 2.79\,\text {MeV}. \end{aligned}$$

BNCT is a two-step treatment which relies on the selective accumulation of $^{10}$B within tumour cells. When these boron-loaded cells are irradiated with thermal neutrons, a nuclear reaction occurs (see Eq. 1). Reaction product particles have a very short path length, ensuring that the radiation damage, which occurs in a distance of a few microns, is confined to the tumour cells containing $^{10}$B. Thereby, this local interaction minimises damage to surrounding healthy tissues and causes significant damage to the DNA of the tumour cells. The reaction between $^{10}$B and neutrons results in high linear energy transfer (LET), which is highly effective in killing cancer cells^2,3^. Epithermal neutrons can be moderated to thermal energies (25.3 meV at 293 K) inside tissue, and thus offer significant clinical advantages by enhancing tissue penetration and enabling the treatment of deeply seated tumours while minimising harm to healthy superficial tissues^1^. BNCT has shown promise in treating various cancers, including glioblastoma multiforme, melanoma, and recurrent head and neck malignancies. The recent clinical trials using hospital-based accelerators have paved the way for more extensive clinical applications and randomised trials^4,5^.

Boric acid ($\hbox {H}_3$ $\hbox {BO}_3$, hereafter BA) has an ideal chemical composition to serve as both a neutron absorber and a neutron moderator^1^. The presence of boron-10 in the BA renders it a natural absorber of thermal neutrons, while the hydroxyl (OH) groups provide enough number density of hydrogen to effectively moderate epithermal neutrons to thermal energies in a series of inelastic collisions in a relatively small space^6–8^. Historically, BA emerged among the earliest investigated boron carriers in BNCT due to its inherent low toxicity, affordability, high water solubility, and ease of handling^1,9^. Although initial clinical trials conducted in the mid-20$^{\hbox {th}}$ century revealed limitations primarily attributed to insufficient tumour selectivity^1,9^, renewed interest has surfaced due to advancements in drug delivery techniques and targeted therapies^1,10–13^.

The evolution of boron delivery agents used in BNCT lasts from the 1950s to the present, with a focus on the rapidly advancing third-generation agents. Until now, boron carriers have progressed through three generations. The first generation-boric acid analogues-was phased out due to poor tumour specificity. The second generation, represented by BPA and BSH, withstood the test of time and remains the only agents currently used in clinical practice, particularly in treating locally invasive malignancies such as melanoma, gliomas, and recurrent head and neck cancers.^14^ Now, third-generation boron agents are emerging in diverse and innovative forms, challenging the clinical dominance of BPA and BSH. Aiming for improved therapeutic outcomes, these new agents are exploring integration of diagnosis and treatment, as well as greater functional diversity, opening up new possibilities for BNCT^15^. However, a recent study investigated the potential of BA for BNCT treatment of hepatocellular carcinoma. The findings revealed that BA preferentially accumulates in hepatoma cells and tumour-associated vasculature, highlighting its capacity for selective tumour targeting^16^. Overall, BA remains valuable both as a foundational boron carrier for research benchmarks and as a potential adjunctive therapeutic agent in the evolving landscape of BNCT.

The distinctive planar trigonal geometry of BA, stabilised by intermolecular hydrogen bonding (H-bonding), gives rise to characteristic vibrational modes that have been extensively studied using infrared (IR) and Raman spectroscopy^17–25^. However, this accurate characterisation of the vibrational properties is only the first step towards further understanding of nuclear quantum effects (NQEs), such as zero-point energy (ZPE), in solid boric acid. In the case of BA, similarly to other molecules containing lightweight nuclear isotopes^26–29^, NQEs, together with isotope effects (e.g., $^{10}$B vs. $^{11}$B), substantially influence the dynamic and thermodynamic properties. NQEs can affect isotope fractionation behaviour and chemical stability relevant to both geochemical and pharmaceutical applications^30,31^. ZPE alters the magnitude of the Doppler broadening of thermal and epithermal neutrons scattered off and/or absorbed by individual isotopic species, thereby influencing the neutronic performance of molecules such as BA. The boron isotope effect is of crucial importance in the case of the BNCT treatment efficacy. The distinguishing of boron isotopes, particularly $^{10}$B, plays a pivotal role in the success of BNCT by enabling selective targeting. High LET radiation, and effective tumour cell destruction while sparing healthy tissues, has its origin in the $^{10}$B isotope only^2^.

Motivated by these considerations, in this work, we characterise nuclear quantum dynamics in solid BA by measuring its broadband neutronic and $\gamma$ response at the VESUVIO thermal-to-epithermal neutron station, located at the ISIS Neutron and Muon Source in Oxfordshire, UK^28,29^. VESUVIO allows simultaneous measurements using thermal-to-epithermal neutron transmission (NT), neutron diffraction (ND), nuclear isotope mass-selective epithermal neutron Compton scattering (NCS), and resonant neutron absorption using neutron resonance capture analysis (NRCA), neutron resonance transmission analysis (NRTA), and prompt gamma activation analysis (PGAA). Taken together, these techniques enable precise determination of average nuclear structure, local nuclear quantum dynamics, and isotopic and elemental composition^28,29^. We use this unique combination of experimental techniques to provide a robust framework for benchmarking high-level *ab initio* simulations of solid BA against neutron observables that depend on thermal and epithermal neutron energies.

Finally, using the multi-technique approach, we obtain limits of detection (LOD) and quantitation (LOQ) of boron in BA in an isotope-selective manner by analysing the magnitudes of the boron recoil peaks recorded in neutron Compton scattering and shapes of $\gamma$ spectra accompanying resonant neutron absorption on boron recorded using the PGAA technique. Moreover, the analysis of the asymptotic (epithermal) parts of the neutron transmission curves recorded in BA on VESUVIO allows for the determination of the LOD and LOQ of natural-abundance boric acid.

By integrating advanced neutron techniques with computational modelling, this work establishes a robust methodological framework for future pilot studies of neutronic performance of boron delivery systems in BNCT and boron-containing neutron moderators for broader applications in medical and nuclear physics.

## Results

### Crystal structure from *ab initio* modelling

**Fig. 1:**
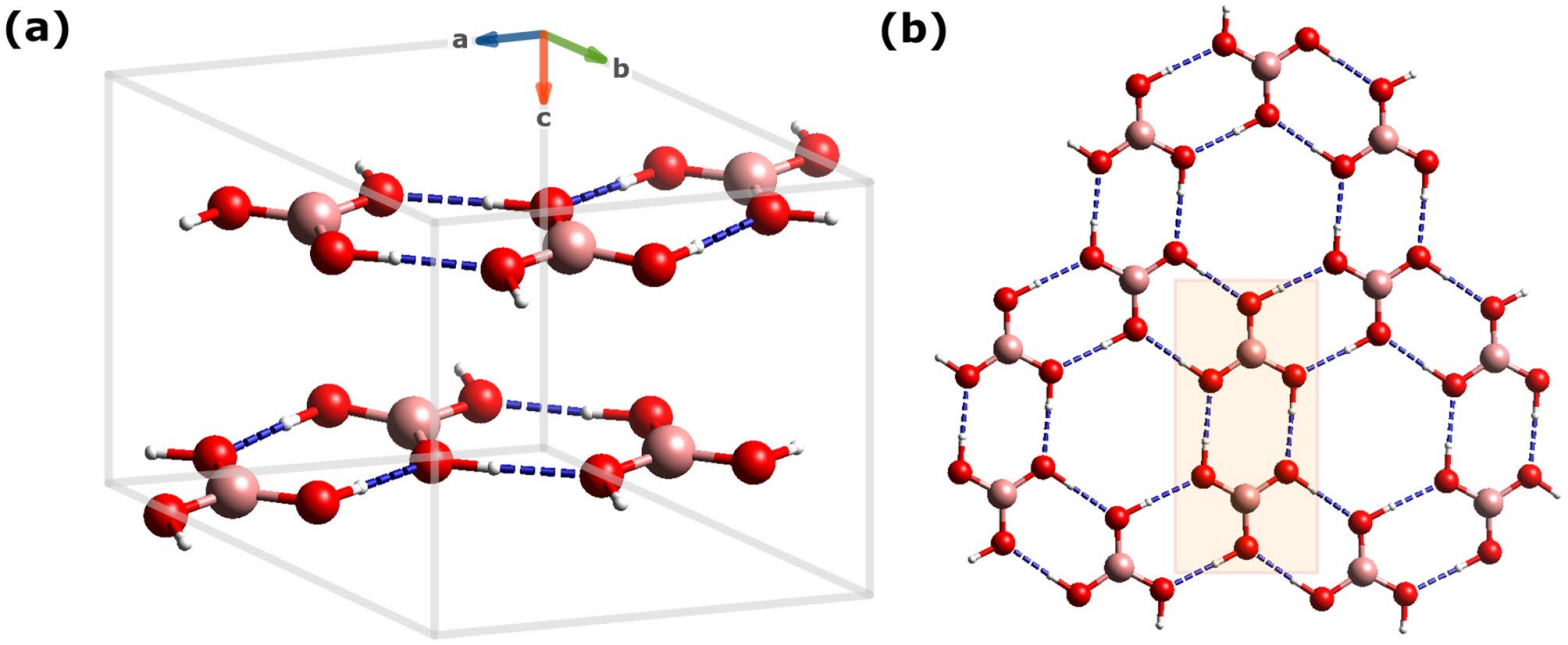
Ball-and-stick representations of the triclinic phase (*P*-1) of BA, with the atomic structure optimised using plane-wave DFT and the PBE-TS functional at 0 K. (**a**) View of the unit cell showing the layered arrangement and H-bonding network along the crystallographic axes. (**b**) Top-down view highlighting the in-plane H-bonded hexagonal motifs. The shaded area marks the asymmetric unit. Atoms are colour-coded: hydrogen (white), boron (pink), and oxygen (red).

Density functional theory (DFT) optimization was carried out on the crystal structure of the triclinic phase (*P*-1) of BA to obtain a mechanically stable configuration (see Fig. 1), confirmed as a minimum on the potential energy surface (PES) by the absence of imaginary modes in the subsequent vibrational analysis. The PBE-TS functional, which incorporates pairwise dispersion corrections, was selected to ensure consistency with previous studies employing similar computational approaches^32^. Its native implementation within the linear-response framework for harmonic lattice dynamics (HLD) calculations in CASTEP^33^ further motivated this choice. The crystal structure of the triclinic form comprises antiparallel ($\hbox {H}_{3}$ $\hbox {BO}_{3}$)$_{2}$ dimers arranged in H-bonded, honeycomb-like layers within the *a-b* plane (see Fig. 1b), which are stacked along the *c*-axis to form the three-dimensional crystal lattice. The resulting triclinic unit cell (see Fig. 1a) has lattice parameters *a* = 6.981 Å, *b* = 6.968 Å, and *c* = 6.418 Å, and cell angles $\alpha$ = 87.37$^{\circ }$, $\beta$ = 105.15$^{\circ }$, and $\gamma$ = 120.22$^{\circ }$. These values, along with internal coordinates, are consistent with other experimental observations of the *P*-1 phase of BA (see Table 1 and section S1 of the SI). A comparison between low-temperature data from Gajhede and co-workers (105 K)^34^ and room-temperature X-ray and neutron measurements^35,36^ reveals an anisotropic thermal expansion. The most pronounced increase occurs along the stacking direction (*c*-axis; +3.62$\%$). The orthogonal *a* and *b* axes, lying within the H-bonded planes, expand more modestly by +0.28% and +0.24%, respectively. The estimated volumetric expansion between 105 K and 300 K is approximately +4.05$\%$, based on the product of the lattice parameters. Theoretical calculations predict a nearly isotropic expansion of approximately +1.3% along all three lattice vectors between 0 K and 300 K, yielding a volumetric increase of about 4.4%, effectively capturing the overall magnitude of thermal expansion. These values are obtained by comparing the static DFT geometry at 0 K with the time-averaged unit cell parameters extracted from fixed-shape, constant-pressure, ambient-temperature *ab initio* molecular dynamics (AIMD) simulations.

**Table 1:** Comparison of the unit cell parameters for the triclinic form (*P*-1) of BA according to plane-wave DFT calculations (PBE-TS) and selected references^34–37^. The DFT-MD simulations at 300 K were performed in the NPT ensemble on a 2$\times$2$\times$2 supercell, with the reported values taken as averaged unit-cell parameters extracted from the trajectory.

Source	Temp. (K)	Method	*a* (Å)	*b* (Å)	*c* (Å)	$\alpha$ ($^{\circ }$)	$\beta$ ($^{\circ }$)	$\gamma$ ($^{\circ }$)
This work (PBE-TS, 0 K)	0	Static DFT	6.981	6.968	6.418	87.37	105.15	120.22
This work (PBE-TS, 300 K)	300	DFT-MD	7.070	7.058	6.499	87.37	105.15	120.22
Craven & Sabine (D_3_^11^$\hbox {BO}_{3}$)^36^	300	Neutron	7.038	7.051	6.575	92.58	101.17	119.83
Gajhede et al.^34^	105	X-Ray	7.019	7.035	6.347	92.49	101.46	119.76
Wu et al.^37^	100	X-Ray	7.018	7.036	6.347	92.48	101.42	119.76
Wu et al.^37^	296	X-Ray	7.032	7.045	6.574	92.51	101.19	119.81
Zachariasen et al.^35^	300	X-Ray	7.039	7.053	6.578	92.58	101.17	119.83

### Vibrational properties

**Fig. 2:**
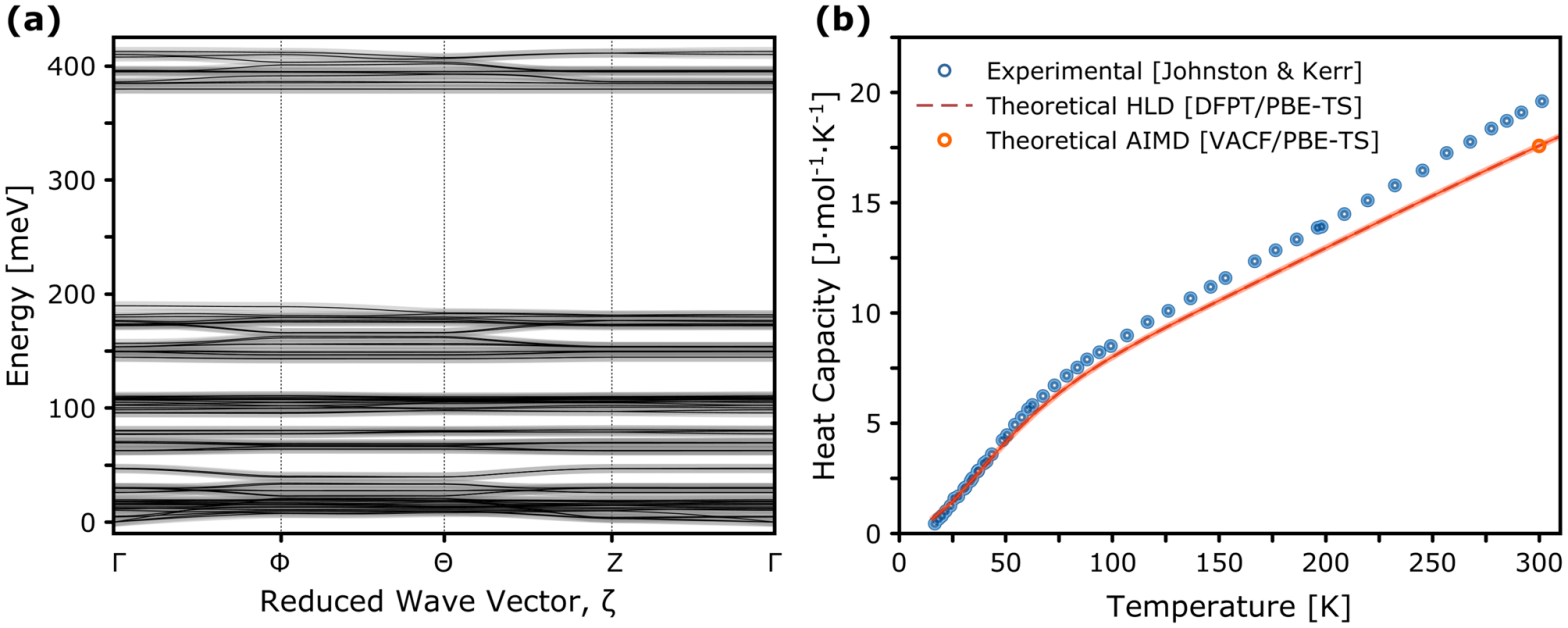
(**a**) Phonon dispersion relations of the triclinic phase (*P*-1) of BA according to DFPT HLD utilising the PBE-TS exchange-correlation functional. The phonon branches are plotted along high-symmetry paths in the Brillouin zone. (**b**) Heat capacity as a function of temperature. Experimental constant-pressure specific heat $C_P$ data (blue circles) from Johnston and Kerr^38^ are compared with the theoretical predictions for the isochoric heat capacity, $C_V$. The theoretical results were obtained from DFPT calculations at 0 K (dashed red line) and from anharmonic AIMD simulations in the microcanonical (NVE) ensemble (orange circles), following the NPT$\xrightarrow {}$NVT equilibration at 300 K.

By accessing the vibrational properties, the first-principles modelling provides further insights into the mechanical stability and thermodynamic properties of the triclinic (*P*-1) phase of $\hbox {H}_{3}$ $\hbox {BO}_{3}$, allowing for direct validation of the accuracy of the computational models against experimental observations. These results are presented in Fig. 2, divided into two panels. Figure 2a shows the phonon band structure calculated along selected high-symmetry directions in the Brillouin zone ($\Gamma$–$\Phi$–$\Theta$–*Z*–$\Gamma$) using density-functional perturbation theory (DFPT). The absence of imaginary frequencies throughout the Brillouin zone confirms the mechanical stability of the structural model at 0 K.

Figure 2b presents the temperature dependence of the specific heat according to Johnston and Kerr^38^, comparing experimental data with theoretical predictions from HLD at 0 K and AIMD at 300 K. The experimental data spans the entire temperature range of interest and exhibits no discontinuities or anomalies, confirming the thermodynamic stability of the triclinic phase throughout the regime relevant to the present work. Overall, good agreement is observed between experiment and theory, although the limitations of HLD in capturing finite-temperature normal mode populations become apparent with increasing temperature, already noticeable from as low as 50 K.

**Fig. 3:**
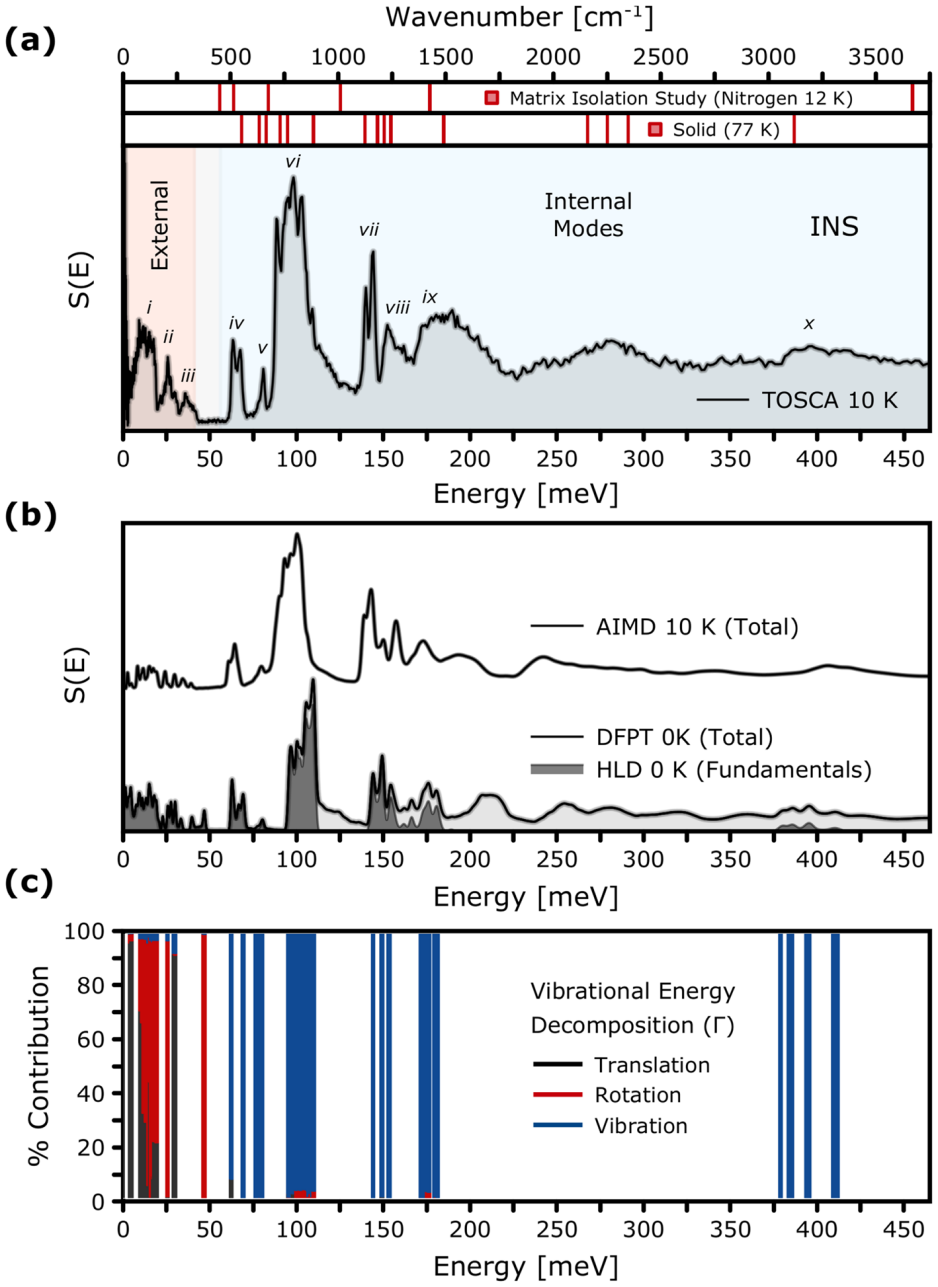
Comparison between **a** the experimental INS spectrum of the triclinic form (*P*-1) of the hydrogenous H_3_^11^$\hbox {BO}_{3}$ by Parker^39^ and **b** the theoretical spectra according to low-temperature HLD (0 K) and AIMD (10 K) simulations. The vertical bars above panel (a) mark the vibrational mode energies identified in previous IR studies: the upper row corresponds to Nitrogen matrix-isolated $\hbox {B(OH)}_{3}$ molecules at 12 K^25^, and the lower row to the IR spectrum of crystalline BA at 77 K^24^. (c) Vibrational energy decomposition of the zone-centre modes according to the DFPT/PBE-TS $\Gamma$-point calculations. Each bar represents the percentage contribution from translational (black), rotational (red), and internal vibrational (blue) motions to a given mode.

The credibility of the vibrational model obtained from the adopted theoretical framework was further validated by comparison with the low-temperature INS spectrum by Parker^39^ (see Fig. 3a). These data were collected at 10 K for a sample enriched to 99% in $^{11}$B to minimise neutron absorption and enhance spectral clarity, using the indirect-geometry TOSCA spectrometer at the ISIS Neutron and Muon Source. These data are freely available for download from the INS Database^40^.

The top panels in Fig. 3b include vertical bars that indicate the positions of IR-active vibrational modes for the matrix-isolated (12 K) and the bulk $\hbox {H}_{3}$ $\hbox {BO}_{3}$ (77 K)^24^. Notable shifts in both stretching (> 375 meV) and bending (ca. 100-175 meV) regions demonstrate the sensitivity of the vibrational response to the local molecular environment, as defined by intermolecular interactions such as hydrogen bonding. The main spectral features predicted by both HLD and AIMD show good agreement in terms of peak positions and relative intensities, supporting the reliability of the adopted theoretical framework. Moreover, negligible differences were found between the INS spectra predicted by HLD for $^{11}$B and $^{10}$B (see section C of the Supplementary Information). Figure 3c shows the decomposition of the phonon eigenvectors, obtained from $\Gamma$-point DFPT calculations, into translational, rotational, and internal vibrational contributions. A detailed analysis of the individual vibrational modes and their spectral assignments is provided in the Supplementary Information (SI) accompanying this work. Based on an extensive examination of the computational data, we provide a detailed list of the observed vibrational energies along with their corresponding mode assignments in Table 2.

**Table 2:** Experimentally observed INS vibrational mode energies for BA. Assignments are based on the HLD mode character from DFPT calculations.

Label	*E*$_{\hbox {INS}}$ [meV]	Assignment	Label	*E*$_{\hbox {INS}}$ [meV]	Assignment	Label	*E*$_{\hbox {INS}}$ [meV]	Assignment
*i*	4.2	Shearing	*iii*	33.8	$\gamma$($\hbox {BO}_3$)	*vii*	139.9	$\delta$(B-O-H)+$\nu$(B-O)
	9.5	Shearing		36.0	$\gamma$($\hbox {BO}_3$)		144.0	$\delta$(B-O-H)+$\nu$(B-O)
	10.5	$\tau$($\hbox {BO}_3$)		39.6	$\gamma$($\hbox {BO}_3$)	*viii*	151.8	$\delta$(B-O-H)+$\nu$(B-O)
	11.6	$\tau$($\hbox {BO}_3$)	*iv*	63.5	$\delta$(O-B-O)		158.6	$\delta$(B-O-H)+$\nu$(B-O)
	12.8	$\tau$($\hbox {BO}_3$)		67.2	$\delta$(O-B-O)	*ix*	174.5	$\gamma$(B-O-H)+$\nu$(B-O)
	15.6	$\tau$($\hbox {BO}_3$)	*v*	77.9	$\gamma$($\hbox {BO}_3$)		187.8	$\gamma$(B-O-H)+$\nu$(B-O)
	18.1	$\tau$($\hbox {BO}_3$)		80.6	$\gamma$($\hbox {BO}_3$)	*$\omega$”	199.1	overtones
*ii*	21.1	$\nu$(O...O)	*vi*	88.6	$\gamma$(B-O-H)		239.0	overtones
	24.4	$\nu$(O...O)		94.1	$\gamma$(B-O-H)		278.5	overtones
	25.9	$\nu$(O...O)		98.4	$\gamma$(B-O-H)		350.7	overtones
	27.4	$\nu$(O...O)		102.9	$\gamma$(B-O-H)	*x*	391.6	$\nu _{\text {asym}}$(O-H)
	29.6	$\nu$(O...O)		109.1	$\gamma$(B-O-H)		412.4	$\nu _{\text {sym}}$(O-H)

*$\omega ''$ , overtones and combination bands; $\nu$, stretching; $\delta$, in-plane bending (scissoring/rocking); $\gamma$, out-of-plane deformation; $\tau$, librational (twisting) motion

### Neutron Compton scattering

**Fig. 4:**
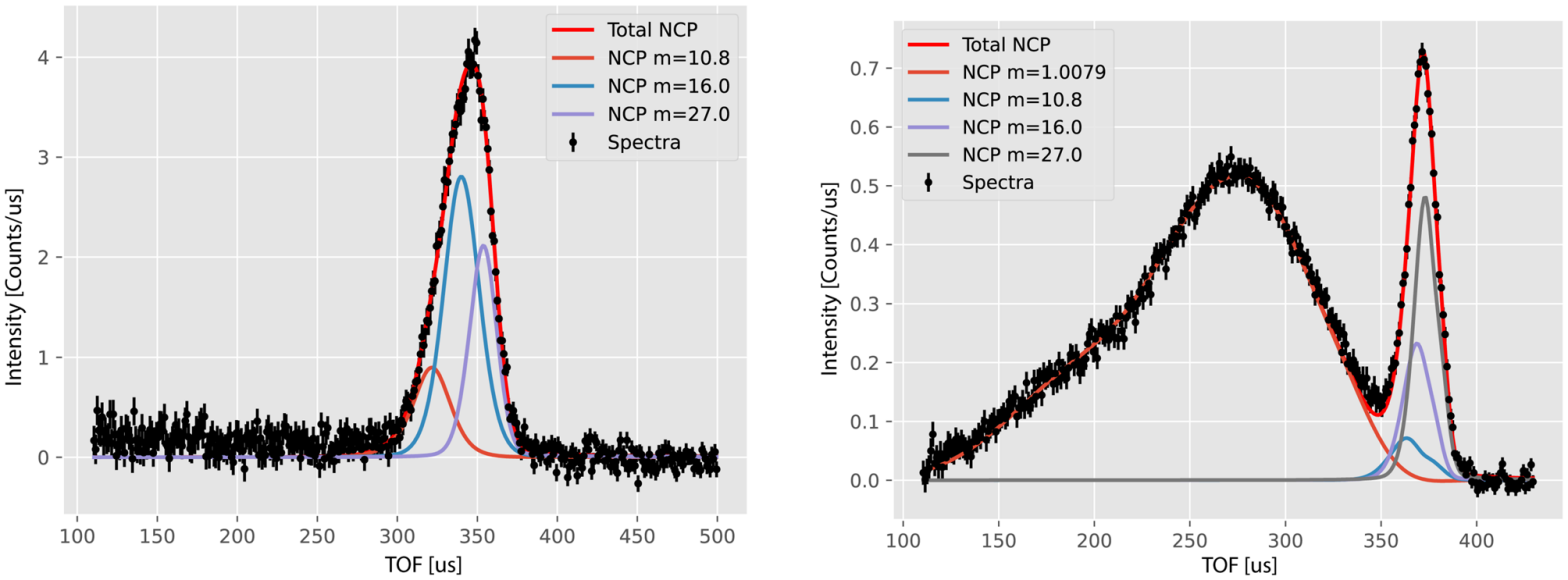
The powder BA sample of the total mass of 2.299 g. Sums (over the entire backward and forward scattering detectors, for the left and right panels, respectively) of recorded NCS TOF spectra are shown as solid black points with error bars. The sums of the fitted recoil lines for individual atomic species are shown as solid lines. See text and figure captions for details.

The NCS spectra of each boric acid sample were recorded and fitted in the time-of-flight (TOF) domain sequentially (on a detector-by-detector basis). In each spectrum, the isotopic mass-resolved nature of the NCS technique was clearly manifested by a sequence of recoil peaks. In forward scattering spectra, the hydrogen recoil peaks were always recorded at the lowest TOF values (in the 100 to 350 µs region), followed by the boron recoil peaks (recorded around ca. 360 µs), the oxygen recoil peaks (recorded around ca. 370 µs), and aluminium container recoil peaks (recorded around ca. 380 µs). Due to the kinematics of the neutron Compton scattering, the centres of all recoil peaks moved with the scattering angle (detector number), with the hydrogen recoil peak centres moving the most from the lowest TOF region (at high scattering angles) towards the region around 270 µs, followed by the boron, oxygen, and aluminium container recoil peaks. In the backscattering, the hydrogen recoil peaks were missing due to the kinematic constraints of Compton scattering (scattering off hydrogen can only be observed in forward scattering angles), and the backscattering spectra were composed only of boron (recorded around ca. 330 µs), oxygen (recorded around ca. 340 µs), and aluminium container recoil peaks (recorded around ca. 370 µs). For clarity, only the sums (over the entire backward and forward scattering detectors, respectively) of recorded TOF spectra and fits were plotted. An example of such a plot is shown in the left (right) panel of Fig. 4 for the NCS data recorded in backscattering (forward scattering) for the sample of the total mass of 2.299 g.

**Fig. 5:**
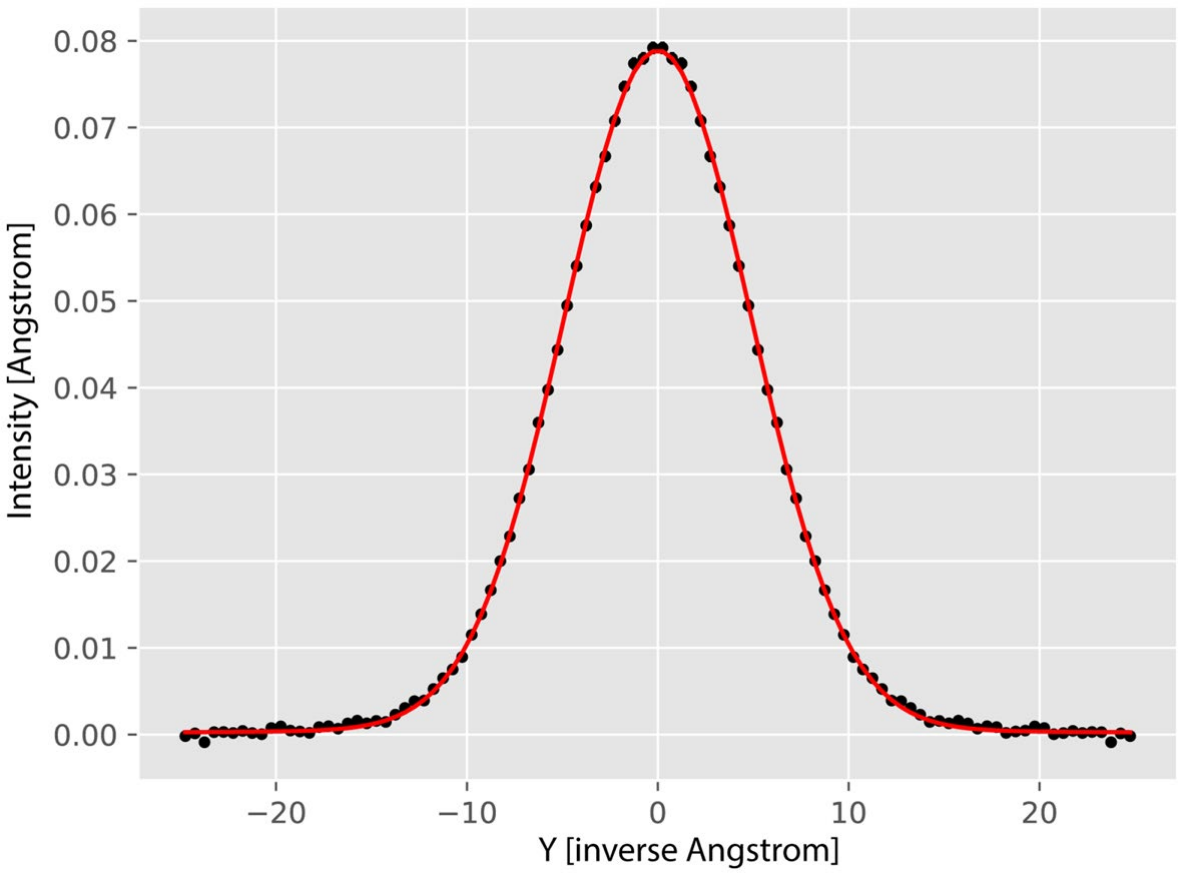
Powder sample of BA of the total mass of 2.299 g. The longitudinal proton momentum distribution focused over all forward scattering detectors (black points and error bars) fitted by a Gaussian profile convoluted with the total instrument resolution function for protons focused in the forward scattering domain (solid red line). See text for details.

In the data recorded by the backscattering detectors, the recoil peaks of boron, oxygen, and the aluminium container are more distinctly separated in the TOF domain compared to forward scattering, owing to the significantly larger momentum transfer from neutrons. It is for that reason that - within the widely accepted NCS data treatment protocol^29,41–48^- the NMD widths of the isotopic species heavier than hydrogen are first fitted in backscattering. The NMD peak widths are then averaged over all backscattering detectors, and the averaged NMD width values together with the standard deviations are obtained. Then, the averaged NMD widths values are fixed in the fitting of the forward scattering data to improve the separation of the proton recoil peaks, which ultimately are transformed into the proton longitudinal momentum distribution domain (see Fig. 5). The values of the proton NMD widths and their standard deviations are then obtained from fitting the proton NMDs focused in this domain.

Neutron Compton scattering enables the assessment of isotope effects on local nuclear dynamics^28,29,43,45,46,49–53^. In the case of hydrogen and lithium, the method enables direct separation of recoil peaks of individual isotopic species^28,29,43,45,46,52,53^, while in the case of heavier atoms like boron, the isotope effects can be observed in the form of broadening of the recoil peaks^49–51^. In this latter case, within the harmonic approximation, the experimental NMD width values for isotopes of mass *M* and $M'$, ($\sigma _{HLD}(M)$ and $\sigma _{HLD}(M')$, respectively) can be assumed (within a mean-field type of approach, where the quantum description of individual vibrational modes is replaced with a single effective quantum harmonic oscillator) to scale according to $\frac{\sigma _{HLD}(M)}{\sigma _{HLD}(M')}=(\frac{M}{M'})^{1/4}$ ^43^, while the values of the experimental kinetic energies, $E_{k,M}$, according to $\frac{E_{k,M}}{E_{k,M'}}=(\frac{M'}{M})^{1/2}$ ^43^. An example of such a procedure applied to the experimental data of the recoil peak of boron is shown in Section A of the Supplementary Information. When the atom-projected VDoSes are simulated within the harmonic approximation, one can model the isotope effects by replacing *M* with *M* in the dynamic matrix, recalculating the phonon dispersion relations and apVDoSes without solving the electronic problems from scratch, and then, using Eq. 5, recalculating the values of the NMD widths and nuclear kinetic energies ^29,43,50,51^. Additionally, one can compare the values of $\sigma _{HLD}(M)$ and $E_{k,M}$ obtained using the mean-field and dynamic matrix approaches. Section B of the SI shows the apVDoSes of H, B and O in H_3_^11^$\hbox {BO}_{3}$ and H_3_^10^$\hbox {BO}_{3}$ obtained following such a procedure. It is worth noting that a similar procedure is not possible within the AIMD simulation scheme, which requires separate NPT, NVT, and NVE simulations for each isotopic species and is outside of the scope of this work. Tables 3 and 4 compare the experimental values of the widths of nuclear momentum distributions and kinetic energies of individual atomic species, present in the powder sample of BA of the total mass of 2.299 g, with predictions obtained from *ab initio* harmonic lattice and molecular dynamics simulations. In the case of boron, the isotope effect is assessed using the mean-field approach for experimental data and the mean-field and dynamic matrix approaches for the HLD predictions. The secondary isotope effects (i.e., changes in the values of $\sigma _{HLD}(M)$ and $E_{k,M}$ of hydrogen and oxygen due to different boron isotope masses) have been calculated to be negligible and are not listed in the tables.

In determining the limits of detection and quantitation of natural-abundance boron, we followed a protocol inspired by our NCS work on melamine^48^. The absolute values of the sums of integrated boron recoil peak intensities recorded by all backscattering detectors on VESUVIO were calculated as a function of the boron mass in BA samples of different masses. The sensitivity parameter, *S*, was then calculated using the following formula^42,48^:

2 $$\begin{aligned} S = \frac{[\text {boron in sample mass - boron in blank mass}]}{[\text {boron in sample counts - blank counts}]} \end{aligned}$$

An empty aluminium sample container was chosen as the blank, with both the blank counts and the boron mass in the blank set to zero. The boron mass in the BA sample of mass 2.298 g is 0.4018 g. The total integral intensity of the boron recoil peak for this sample was 33.5 counts. Thus, the value of *S* is 0.012 [g/counts]. Using the value of *S* and the standard deviation of the blank measurement, one obtains the value of the LOD of $S \times 3 \times 1 = 3 \times S$ and the value of the LOQ of $S \times 10 \times 1 = 10 S$. Using these two relations, the values of the LOD and LOQ are 0.036 and 0.12 g, respectively. Assuming that the scattering intensity of a recoil peak of mass of an isotopic species of *M* is proportional to the value of its total bound scattering cross section (see Eq. 11), the LOD and LOQ values for $^{10}$B are 0.061 and 0.20 g, respectively, while their counterparts for $^{11}$B are 0.032 and 0.11 g.

**Table 3:** The experimental values of the widths of nuclear momentum distributions of individual atomic species, present in the powder sample of BA of the total mass of 2.299 g, with predictions obtained from *ab initio* harmonic lattice and molecular dynamics simulations. In the case of boron, the isotope effect is assessed using the mean-field approach for experimental data and the mean-field and dynamic matrix approaches for the HLD predictions. NMD width values are given in units of Å$^{-1}$. See text for details.

Nucleus	HLD prediction (mean field)	HLD prediction (dynamic matrix)	AIMD prediction	Fit (mean field)
H	–	5.04	4.98	4.78 ± 0.02
B	–	12.54	12.45	9.60 ± 0.9
$^{10}$B	12.30	12.27	–	9.42 ± 0.9
$^{11}$B	12.60	12.57	–	9.64 ± 0.9
O	–	12.59	12.42	11.4 ± 1.1

**Table 4:** The experimental values of the kinetic energies of individual atomic species, present in the powder sample of BA of the total mass of 2.299 g, with predictions obtained from *ab initio* harmonic lattice and molecular dynamics simulations. In the case of boron, the isotope effect is assessed using the mean-field approach for experimental data and the mean-field and dynamic matrix approaches for the HLD predictions. The kinetic energy values are given in units of meV. See text for details.

Nucleus	HLD prediction (mean field)	HLD prediction (dynamic matrix)	AIMD prediction	Fit (mean field)
H	–	158.0	154.2	142.1 ± 1.2
B	–	91.3	90.0	53.5 ± 10.0
$^{10}$B	94.8	94.4	–	55.6 ± 10.4
$^{11}$B	90.5	90.1	–	53.0 ± 9.9
O	–	62.1	60.4	50.9 ± 9.8

### Neutron transmission and total neutron cross-section

**Fig. 6:**
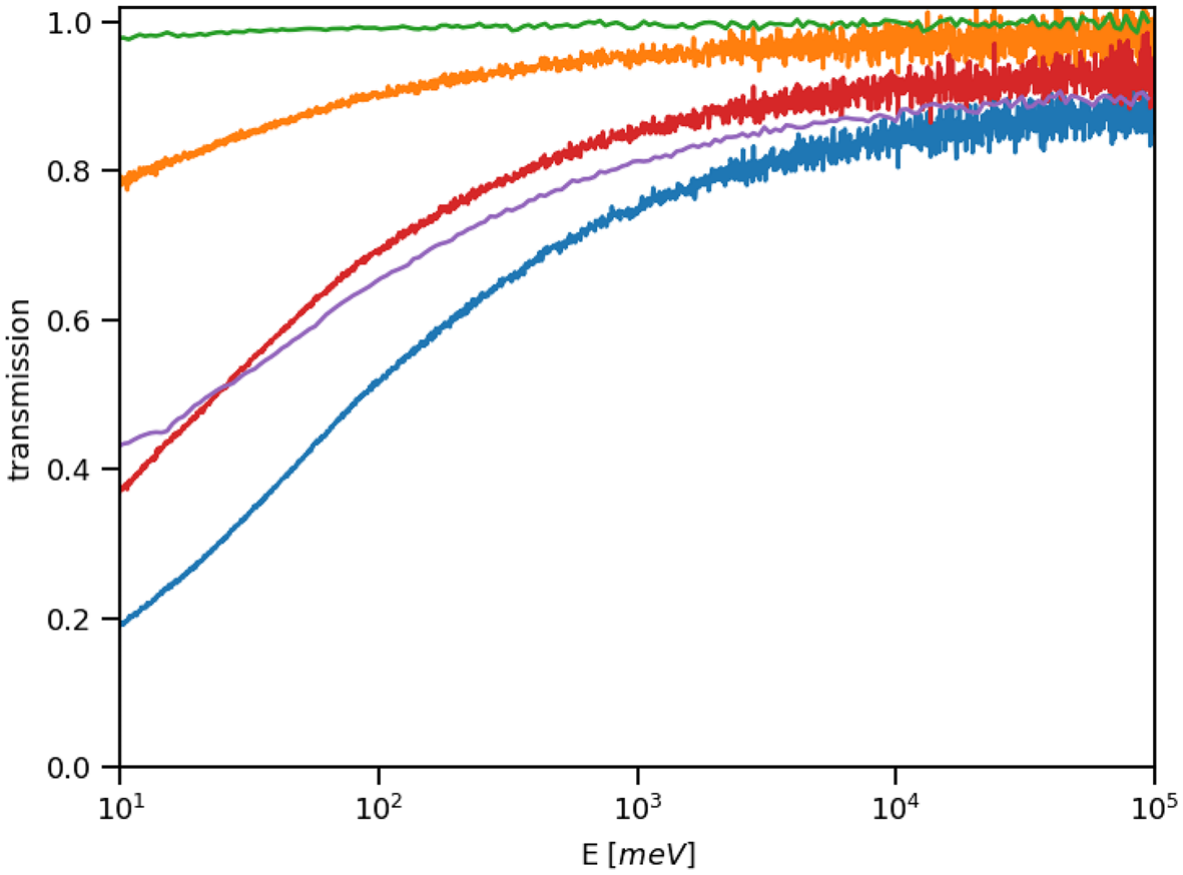
Incident neutron energy-dependent transmission curves of BA samples with decreasing masses. For each curve, the average sample transmission and scattering power were calculated from data recorded in the incident neutron energy range of 10-100 eV. See text for details.

**Fig. 7:**
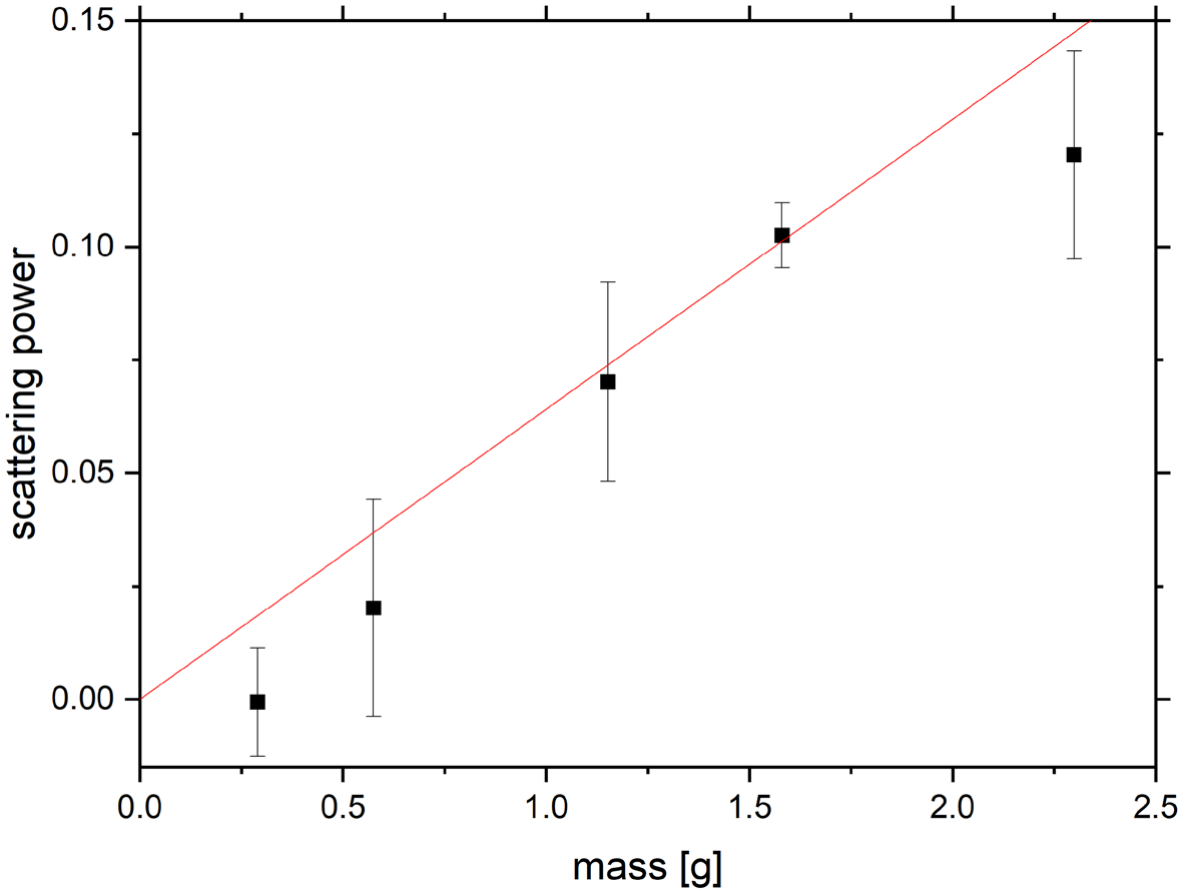
The sensitivity plot for the detection of natural abundance boron in powder samples of BA using incident neutron energy-dependent sample transmission. See text for details.

The results of a series of neutron transmission experiments on a set of dry powder samples of BA with increasing mass, performed on the VESUVIO beamline, are shown in Fig. 6. Following the methodology already presented in our work on the limits of detection of illicit substances on the VESUVIO beamline^48^, the plateau regions of transmission curves at the epithermal incident neutron energies, 10-100 eV, were chosen to calculate the average sample transmission and scattering power (see Fig. 7). Due to the limitations of the experimental protocol, experiments of different durations (and thus characterised by different signal-to-noise ratios) were performed for BA samples of varying masses.

The sensitivity parameter, S, and the values of LOD and LOQ were obtained using data shown in Figs. 6 and 7, following the methodology from our previous NCS work^42,48^. The sample scattering power was defined as one minus the value of the sample transmission averaged over the region of incident neutron energies between 10 and 100 eV. The linear regression analysis of the data shown in Fig. 7 yielded the value of S = 1/(0.064 ± 0.007) g = 15.6 ±1.7 g. The standard deviation of the scattering power of the empty aluminium container, chosen as a blank, was 0.003. Thus, the value of the LOD was $(15.6 \pm 1.7) \times 3 \times 0.003 = 0.141 \pm 0.015$ g and the value of the LOQ was $(15.6 \pm 1.7) \times 10 \times 0.003 = 0.469 \pm 0.051$ g.

**Fig. 8:**
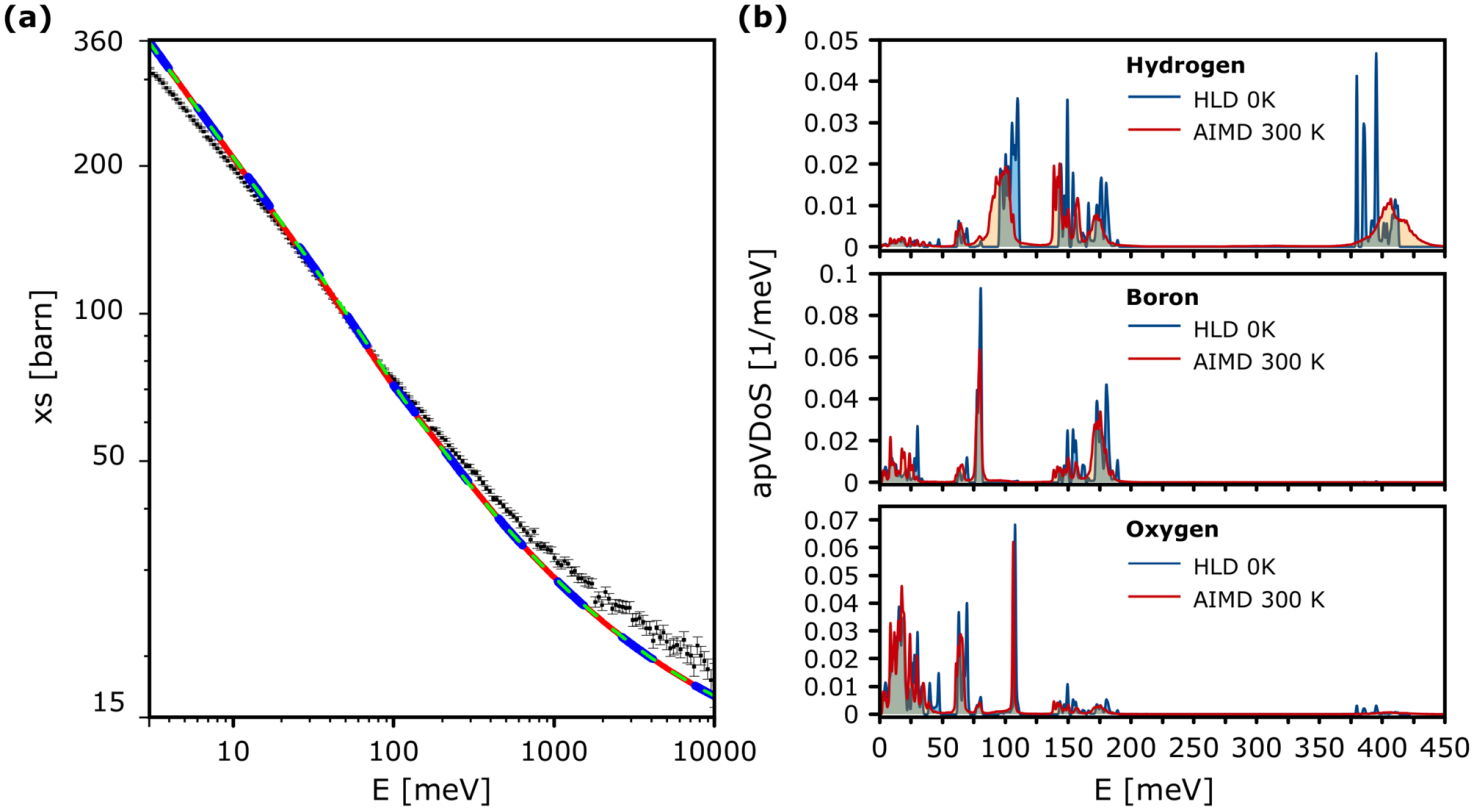
Panel a: the comparison of the experimental total neutron cross-section, obtained from the transmission measured for the BA sample of mass 1.150 g (solid black markers with error bars), with simulated total neutron cross-sections obtained employing the multi-phonon expansion (MPE) method using the VDoSes of natural abundance boron, oxygen and hydrogen obtained from the HLD (the dashed blue line), AIMD (dashed red line) and AFGA (dashed green line) approximations. Panel b: the apVDoSes of natural abundance boron, oxygen, and hydrogen, obtained from the HLD simulations at 0 K (solid blue lines) and their counterparts from the NVE AIMD simulations at 300 K (solid red lines).

Figure 8a compares the experimental total neutron cross-section, obtained from the transmission measured for the BA sample of mass 1.150 g, with simulated total neutron cross-sections obtained employing the multi-phonon expansion (MPE) method using the apVDoSes of natural abundance boron, oxygen and hydrogen obtained from the HLD, AIMD and AFGA approximations. The apVDoSes are shown in Fig. 8b.

### Prompt gamma activation analysis

**Fig. 9:**
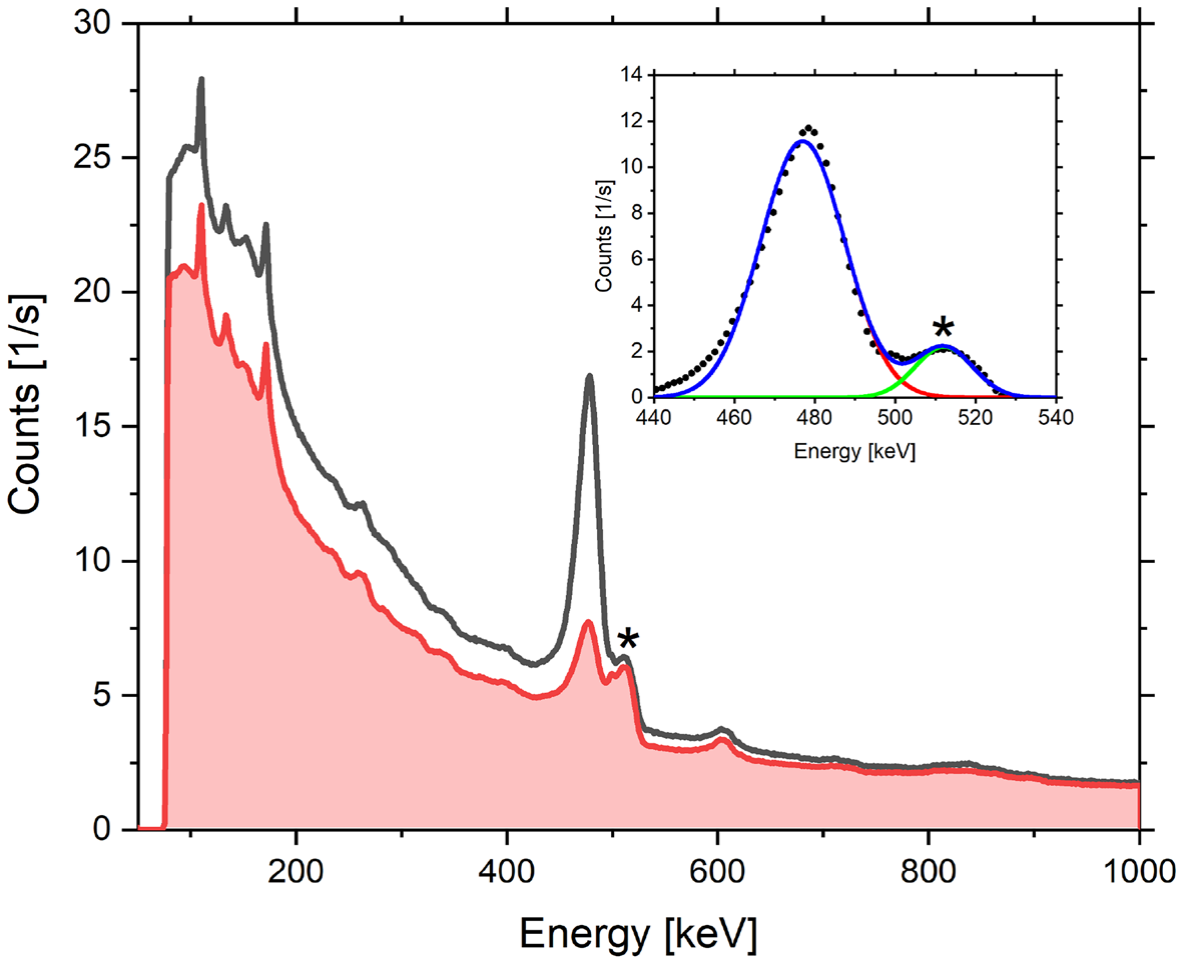
The raw measured $\gamma$ spectrum of the boric acid sample of mass 0.575 g (solid black line), together with the total $\gamma$ background present in the VESUVIO blockhouse during the neutron data acquisition (solid red line and shaded red area). The inset shows the processed spectrum after the subtraction of the background. The spectrum consists of a line at 478 keV $\gamma$ energy due to resonant neutron absorption on boron, fitted by a Gaussian profile represented by a solid red line, and a line due to the annihilation photons at 511 keV (marked by an asterisk), fitted by a Gaussian profile represented by a solid green line, that originates from pair production of high-energy $\gamma$ rays.

Figure 9 shows the measured $\gamma$ spectrum of the boric acid sample of mass 0.575 g, together with the total $\gamma$ background present in the VESUVIO blockhouse during the neutron data acquisition. The region of interest of $\gamma$ energies for the determination of the limits of detection and quantitation of boron using the PGAA technique is between 400 – 550 keV. In this energy range, the peak at 478 keV due to neutron capture on boron partially overlaps with a peak due to the annihilation photons at 511 keV, which originates from pair production of high-energy $\gamma$ rays (see the inset of Fig. 9)^54^. Additionally, the peak at 478 keV is Doppler-broadened by the motion of the $^{7}$Li nuclei^54^.

**Fig. 10:**
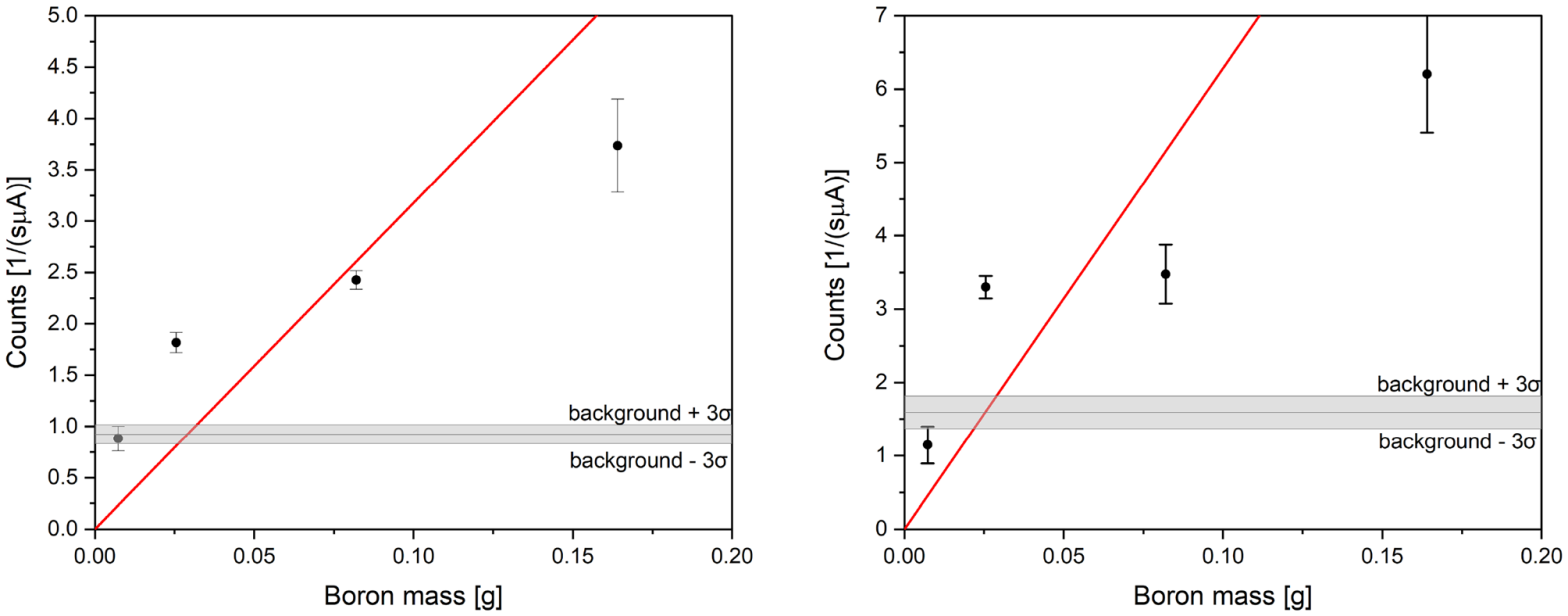
The sensitivity curves used for the determination of the values of the LOD and LOQ for boron in solid boric acid recorded using the PGAA technique. The curves were obtained by plotting the integral intensities of the peaks at 478 keV $\gamma$ energy fitted using Gaussian (left panel) and Lorentzian (right panel) peak profiles versus sample mass. The lowest integral intensities, fitted for samples of boric acid containing 7.3 mg of boron, were within three standard deviation values of the integral intensities of the background, shown as horizontal, grey-shaded areas.

Figure 10 shows the sensitivity curves used from the determination of the values of the LOD and LOQ for boron in solid boric acid recorded using the PGAA technique. The curves were obtained by plotting the integral intensities of the peaks at 478 keV $\gamma$ energy fitted using Gaussian (left panel) and Lorentzian (right panel) peak profiles against the mass of boron in BA samples of different masses. As in the case of LOD and LOQ determination from neutron data, the data point collected for the BA sample with the biggest mass was excluded from the regression analysis. In the neutron data analysis, this data point deviates from the linear trend visible for samples of lower masses, most likely due to multiple scattering in the relatively thick sample. The sample thickness may also cause attenuation of $\gamma$ rays, which renders the quantitation of boron in the BA sample impossible. The lowest integral intensities, fitted for samples of boric acid containing 7.3 mg of boron, were within three standard deviation values of the integral intensities of the background, shown as horizontal, grey-shaded areas.

For the boron gamma peak modelled as a Gaussian, the sensitivity parameter, obtained as an inverse of the slope of the linear regression (solid red line in the left panel in Fig. 10), $S = 1/(31.76 \pm 6.98) = 0.0315 \pm 0.0069~[\textrm{g}/\textrm{counts}]$. The standard deviation of the blank measurement (equal to one-sixth of the thickness of the greyed-out area in the plot, indicating the uncertainty of the background level) was 0.031 counts. Thus, the $\textrm{LOD} = 3 \times (0.0315 \pm 0.0069) \times 0.031 = 2.93 \pm 0.64$ mg, and the $\textrm{LOQ} = 9.77 \pm 2.14$ mg. In the case of the boron gamma peak modelled as a Lorentzian, the sensitivity parameter $S = 1/(62.8 \pm 22.7) = 0.0159 \pm 0.0058~[\textrm{g}/\textrm{counts}]$. The standard deviation of the blank measurement was 0.075 counts. Thus, $\textrm{LOD} = 3 \times (0.0159 \pm 0.0058) \times 0.075 = 3.58 \pm 1.31$ mg, and $\textrm{LOQ} = 11.9 \pm 4.4$ mg.

## Discussion

The experimental and theoretical data presented above serve as a foundational basis for discussing the key findings of this study. To build upon these insights, we begin with a detailed assessment of the performance of our theoretical framework. This computational framework was rigorously tested against available experimental benchmarks and subsequently utilised to enhance the analysis through a multitechnique approach involving the VESUVIO spectrometer. By integrating theoretical predictions with advanced neutron spectroscopy, we aim to provide a comprehensive and robust atomistic picture of crystalline BA. In particular, the structural properties appear to provide a fundamental basis for interpreting the nuclear dynamics of BA and its related thermophysical behaviour, guiding the direction of our analysis throughout the study. Moreover, the molecular dynamics component of our theoretical framework is of great importance in the context of the studies of potential new boron carriers and their efficiency in BNCT treatment^15^.

The crystal structure of $\hbox {H}_{3}$ $\hbox {BO}_{3}$ has been the subject of extensive investigation over the past century, as one of the first hydrogen-bonded molecular crystals to be examined by X-ray diffraction.^35,55^. The initial X-ray diffraction insights by Zachariasen revealed that BA crystallises in a triclinic layered structure belonging to the *P*-1 space group, with four molecules per unit cell. Subsequent studies, including neutron diffraction on a perdeuterated D_3_^11^$\hbox {BO}_{3}$ single-crystal specimen, enabled a more precise determination of light atom positions. The structural picture reveals that the layers form a weakly buckled, double-sheeted motif arranged in an antiparallel AB-type stacking pattern (hereafter $\hbox {H}_{3}$ $\hbox {BO}_{3}$-2A; see Fig. 1a). An alternative, trigonal $\hbox {H}_3$ $\hbox {BO}_3$-3T (space group *P*$\hbox {3}_2$) polytype has also been identified by Shuvalov and Burns^56^. The trigonal phase features layers of H-bonded BA molecules closely resembling those in the triclinic form. In both structures, the layers are held together by van der Waals (vdW) interactions, with the key difference lying in their stacking patterns along the crystallographic c-axis (see Fig. 1a). Although the in-plane arrangement of the triclinic layers in the $\hbox {H}_3$ $\hbox {BO}_3$-2A form approximates a trigonal lattice, the H-bonded layers are offset along the stacking direction. In the trigonal $\hbox {H}_3$ $\hbox {BO}_3$-3T phase, the molecular sheets follow an ABC stacking sequence, while in $\hbox {H}_3$ $\hbox {BO}_3$-2A, the sequence follows an AB-type pattern. The triclinic phase is the most prevalent under ambient conditions, whereas the trigonal form, which is slightly more stable thermodynamically, can be obtained through specialised preparation methods^56^.

In both the 2A and 3H polytypes, each stacked sheet comprises planar $\hbox {H}_{3}$ $\hbox {BO}_{3}$ units with nearly $\textrm{C}_{3\textrm{h}}$ symmetry, interconnected by a network of moderately strong hydrogen bonds. Recent computational work shows that the $\textrm{C}_{3\textrm{h}}$ minimum is energetically favoured over alternative conformers such as $\textrm{C}_\textrm{s}$, which lies $\sim$4.1 kcal/mol higher and presents slight bond length asymmetry due to electronic hyperconjugation effects ^57^. These threefold-symmetric units can be described as molecular triskelions, each featuring threefold rotational symmetry ($\textrm{C}_3$) and a horizontal mirror plane ($\sigma _\textrm{h}$), with three outward-extending O-H arms that engage in H-bonding (see Fig. 1b)^58^. The symmetry elements of the layered structure depicted in Fig. 1b^58^ correspond to the point group $\textrm{C}_{6\textrm{h}}$ ^24^. Within this framework, boron atoms occupy equivalent positions arranged symmetrically around each hexagonal axis. The ‘layer unit cell’ depicted in Fig. 1b^58^ comprises two such molecules positioned symmetrically with respect to an inversion centre, forming a local dimeric motif with $\textrm{C}_{2\textrm{h}}$ symmetry. The molecular structure is described by average B-O bond lengths of approximately 1.37 Å and O-H distances near 0.97 Å, as consistently determined by room-temperature neutron diffraction and corroborated by high-level quantum chemical calculations^57^ and our present work (see Fig. S1 and Table S1)

Focusing on the triclinic $\hbox {H}_{3}$ $\hbox {BO}_{3}$-2A polytype, individual $\hbox {B(OH)}_{3}$ molecules organise into planar layers stabilised predominantly by hydrogen bonding, with O$\cdots$O separations of approximately 2.72 Å. The interlayer spacing is around 3.2 Å^34^, and *ab initio* calculations indicate that the binding energy between layers is roughly one-third of the intralayer interaction energy^59^, highlighting the dominant role of H-bonding within the layers. By comparing the results of our DFT calculations with the experimental structural data compiled in Fig. S1 and Table S1, we observe a marked improvement of finite-temperature simulations in the description of both molecular geometry and intermolecular contacts. Similar interatomic distances and geometry of the H-bonded network in solid BA bear a resemblance to that of hexagonal ice (ice $\hbox {I}_{h}$), particularly in the pseudo-hexagonal arrangement that closely mimics the topology observed in the basal planes of ice $\hbox {I}_{\hbox {h}}$ ^60,61^. However, unlike ice $\hbox {I}_{\hbox {h}}$, where the H-bonding extends in a fully three-dimensional, tetrahedrally coordinated network, BA exhibits discrete molecular layers connected by comparatively weaker interlayer interactions, resulting in a fundamentally two-dimensional structural character. This structural similarity is compelling because the BA layers, unlike the continuous 3D network of ice, serve as a simplified, controllable analogue for dissecting H-bond topology and proton dynamics within the pseudo-hexagonal motifs. Given the structural similarity, this poses a question about the extent to which BA exhibits comparable proton dynamics. In ice $\hbox {I}_{\hbox {h}}$, the low-temperature studies have revealed highly non-trivial proton motion, including collective six-site tunnelling and quantum delocalisation phenomena^62–70^. These effects are closely tied to the frustrated proton-disordered network and the low energy barriers for proton transfer along the H-bonds.

To discern the underlying vibrational dynamics, we first examine the phonon dispersion relations. Figure 2a presents the phonon band structure calculated along the high-symmetry directions of the Brillouin zone with linear-response HLD. The absence of imaginary phonon frequencies throughout the Brillouin zone confirms the mechanical stability of the structural model at 0 K. The phonon band structure exhibits weak dispersion and flattens rapidly, which is consistent with the layered character of the crystal and the dominance of intra-layer over stacking interactions. To date, phonon dispersion relations have not been accessed experimentally for boric acid crystals, and no inelastic neutron or X-ray scattering data are available. Nonetheless, a rich body of spectroscopic and thermophysical studies has been developed over several decades^18–21,23–25,38,71^, providing indirect yet essential insights into the lattice dynamics of the $\hbox {H}_{3}$ $\hbox {BO}_{3}$-2A polytype.

Particularly, the thermophysical data reported by Johnston and Kerr^38^ and Oguni et al.^71^ offer a reliable benchmark for scrutinising the accuracy of the *ab initio* predictions and the estimate of the extent of disorder, which has been debated from the outset^34,35,71–75^. Figure 2b illustrates the temperature dependence of the specific heat based on high-precision adiabatic calorimetry measurements by Johnston and Kerr^38^. The theoretical isochoric specific heat, derived within the harmonic approximation, closely follows the experimental trend across a broad temperature range, though notable deviations emerge already above 50 K. At higher temperatures, the calculated $C_V$ values visibly underestimate the experimental data, owing to neglect of anharmonicity and thermal expansion effects. Moreover, the early experimental work by Johnston and Kerr^38^ was later revisited by Oguni et al.^71^, who extended the calorimetric measurements up to 370 K and re-evaluated the earlier interpretations. This work further revealed a heat capacity anomaly near 290 K, amounting to 3-4$\%$ of the total heat capacity. This anomaly was confirmed for both hydrogenous and perdeuterated specimens and was attributed to a rearrangement of the H-bonding framework. In an attempt to account for mechanical anharmonicity and thermal expansion effects, the specific heat was also estimated from the finite-temperature VDoS obtained via AIMD simulations at 300 K. This allowed us to assess the credibility of the finite-temperature simulations relevant to VESUVIO experiments. As noted in our discussion of Eq. 8, capturing the temperature dependence of the isochoric specific heat ($C_V$) using AIMD would ideally require a series of simulations to compute the total VDoS across a grid of temperatures within the relevant range. However, the use of a single VDoS obtained from an AIMD simulation at only one temperature (here, 300 K) is justifiable only if the VDoS remains largely invariant throughout that temperature interval. For this reason, in Fig. 2b, we report only the $C_V$ value obtained from the AIMD-derived VDoS at 300 K.

The positioning of hydrogen atoms in BA has been a topic of longstanding debate since the initial determination of the boron and oxygen positions via early X-ray diffraction studies by Zachariasen^35^. Later investigations using electron diffraction by Cowley^72^ revealed that hydrogen atoms are displaced from the idealised O–O axis, suggesting positional disorder ^71^. This view was further supported by nonlinear O-H$\cdots$O bonding models proposed by Kume and Kakiuchi^74^, based on NMR data. Using second moment ($\hbox {M}_{2}$) NMR analysis, the authors have demonstrated that the observed linewidths were incompatible with collinear H-bonding geometry. Instead, the data supported a model in which protons are displaced off the O$\cdots$O axis, occupying two symmetry-equivalent sites, thereby reducing dipolar coupling and suggesting positional disorder within the hydrogen bond network. Yet, these conclusions were subsequently challenged by Ibers and Holm^76^. In a later structural reinvestigation, the neutron diffraction measurements were explicitly designed to resolve the hydrogen positions^36^. Although the complete disorder has been ruled out, a partial disordering of the proton position in a double minimum potential was suggested by Oguni et al.^71^. This phenomenon has been associated with a glass transition and interpreted as a relaxation effect arising from the collective redistribution of hydrogen atoms within the stacked molecular sheets. The transition is thought to originate from frozen-in disorder between clockwise and anticlockwise configurations of the H-bonded $\textrm{C}_{6\textrm{h}}$ arrays^24^. In light of these findings, we consider the performance of our AIMD simulations to be excellent in capturing the structural and thermophysical properties. It is important to acknowledge that standard Born–Oppenheimer molecular dynamics (BOMD), as employed in this work, inherently treats the nuclei as classical particles^77^. As a consequence, ZPE contributions are not captured within this framework directly, requiring effective temperature^78^. While BOMD offers valuable insights into thermally activated structural and dynamical processes, its classical nature limits its ability to describe purely quantum effects such as ZPE and nuclear delocalisation.

The vibrational properties of the $\hbox {H}_{3}$ $\hbox {BO}_{3}$-2A polytype have been the subject of a broad spectroscopic investigation. Most importantly, initial insights were provided by Bethell and Sheppard^19^, who reported infrared (IR) spectra in the solid state and discussed assignments based on symmetry arguments. This work was followed by Hornig and Plumb^24^, who extended the analysis to include normal-mode interpretations. Later, Durig et al.^18^ conducted detailed far-IR and Raman measurements to emphasise the low-frequency lattice vibrations and interlayer dynamics. Subsequent studies, such as Broadhead and Newman^21^, extended the analysis towards the thermal decomposition tracking. More recently, Bezerra da Silva et al.^20^ employed similar-quality calculations (CASTEP/PBE-TS) to elucidate vibrational features of the 2A and 3T polymorphs. Here, we further advance this picture by directly confronting our computational predictions with INS measurements, as reported by Parker^39^. Owing to the dominant incoherent scattering cross section of hydrogen atoms, the presented INS spectrum predominantly reflects proton dynamics. Figure 3 compiles the results of experimental INS measurements according to Parker^39^(see Fig. 3a) alongside the computational results from both HLD (0 K) and AIMD (10 K) (see Fig. 3b). The zone-centre phonons are further decomposed in terms of the vibrational energy as presented in Fig. 3c and the S1 (Figs. S2 alongside Table S2). The visualisation of the normal mode displacements is collected in Figs. S3–S13. In addition, the results of low-temperature IR spectroscopy, derived from both matrix isolation and conventional solid-state measurements, are shown as vertical bars above the panel in Fig. 3a, enabling direct comparison with the INS spectrum. Table 2 summarises the full list of experimentally observed INS peaks, along with tentative mode assignments based on the HLD calculations. For visualisation of the corresponding normal modes, see Figs. S3–S14 in the SI.

A quantitative comparison between the IR spectra of the $\hbox {H}_{3}$ $\hbox {BO}_{3}$-2A polytype, as shown in Fig. 3a, measured at 77 K in the solid state and at 12 K under nitrogen matrix isolation conditions, highlights the pronounced influence of intermolecular interactions on the vibrational dynamics. The high-energy O–H stretching region exhibits the most substantial change, where cooperative H-bonding in the solid state results in a broad and intense IR band that is red-shifted by approximately 25–55 meV compared to the matrix-isolated molecule. Interestingly, a further temperature-dependent comparison reveals a slight blue shift of this band upon heating from 77 K to room temperature. Specifically, the main peak in the O–H stretching region shifts from 387 meV to 397 meV, contrary to the behaviour typically expected in H-bonded systems. This contrasts with the red shift observed in H-bonded systems such as ice $\hbox {I}_{\hbox {h}}$, where reduced cooperativity and bond elongation dominate the temperature dependence. While this intriguing effect appears counterintuitive, the observed blue shift may stem from enhanced mode cooperativity within the H-bonded network due to coupling among adjacent molecular units, which can effectively stiffen the vibrational response. Furthermore, it may reflect subtle structural rearrangements within the H-bonding network and nontrivial anharmonic effects^79^. As opposed to optical vibrational spectroscopy, INS enables the observation of all vibrational excitations regardless of optical selection rules and mode symmetry. The INS spectrum shown in Fig. 3a provides a rich hydrogen-projected vibrational response over a broad energy range. Fundamental modes are detected between approximately 175 meV and 375–425 meV, with their intensities decreasing progressively at higher energy transfers. In the upper part of this range, additional features attributed to overtones and combination bands become visible. These contributions are enhanced due to the relatively high momentum transfers associated with the fixed *Q*, *E* trajectory of the TOSCA spectrometer^80^. At this point, we note the presence of a pronounced band near 280 meV, which is not reproduced by the theoretical model and is more likely attributable to a combination band.

While classically-sampled AIMD does not explicitly incorporate ZPE and is therefore subjected to limitations in describing nuclear dynamics at low temperatures, it nevertheless captures certain features of the vibrational behaviour that are inaccessible to HLD. Even at temperatures as low as 10 K, AIMD can offer an improved representation of the vibrational spectrum in specific regimes, particularly for the modes associated with shallow or anharmonic regions of the PES. This improvement stems from the absence of any assumptions regarding the shape of the underlying potential and the natural inclusion of mode coupling in the simulations. While AIMD and HLD yield broadly similar results for well-localised, near-harmonic modes, clear differences emerge in the case of soft torsional or out-of-plane deformations. These differences are evident in the observed shifts of peak centres in the calculated INS spectra using both methodologies. By inspection of Fig. 3b along with Table 2, several key differences are observed between the vibrational signatures predicted by HLD and those obtained via AIMD. These discrepancies are particularly pronounced below 50 meV, between 75–175 meV, and in the high-energy domain spanning 375–425 meV, respectively. In the low-frequency range (< 50 meV), the substantially affected modes can be ascribed to the hydrogen-bridge dynamics (O...O motions). The spectral differences in this range point toward coupling between slow and fast vibrational modes within the hydrogen bond network^81,82^. Additionally, the visible red shifts observed for the bands centred around 100 meV and 150 meV indicate significant anharmonicity in the out-of-plane and in-plane deformation modes, respectively. These effects are largely driven by mode coupling, as evidenced by a subtle blue shift in AIMD spectra even at low temperatures 10 K when compared to HLD predictions. Interestingly, classical AIMD simulations suggest a slight stiffening of the O–H bond, manifested as a frequency upshift of approximately 10 meV in the stretching region. Control tests on the frequency shift associated with the Verlet integrator revealed that finite-time-step errors remain negligible, with an estimated deviation of less than 2 meV at 400 meV. Finally, comparing the computed bond lengths (Table S1) suggests that the 0 K DFT values slightly overestimate H-bond strength when compared to the finite-temperature simulations and the reference neutron diffraction data, which aligns with the blue shift predicted by classically sampled MD-DFT.

As far as the nuclear quantum observables (NMD widths and nuclear kinetic energies) are concerned, the values obtained based on the AIMD simulation performed at 300 K lie systematically closer to experimental values than their counterparts obtained employing apVDoSes from the HLD simulation performed at the limit of zero Kelvin. Due to the Bose-Einstein distribution, at finite temperatures, low-frequency modes are more significantly excited than high-frequency modes, and the low-frequency part of the VDoS dominates the specific heat values. Contrary to the specific heat, in the case of nuclear kinetic energy, the dominating part is due to high-frequency modes, as they carry the largest amounts of nuclear kinetic energy. Thus, the differences between the shapes of the apVDoSes of H, B and O computed using the HLD and AIMD at 300K (see Fig. 8) will contribute more towards the differences between the simulated values of the nuclear kinetic energies of these individual atomic species than towards the difference between the values of the isochoric specific heat predicted by the HLD and AIMD scheme. This explains why, in the case of specific heat, the HLD and AIMD-derived values overlap almost exactly at 300 K, whereas in the case of nuclear kinetic energies, the AIMD-predicted values are closer to their experimental counterparts.

The AIMD-based computational protocol shows improvement over the HLD counterpart due to the inclusion of mode softening/hardening effects induced by temperature on one side, and accounting for the quantum population of vibrational energy levels on the other. However, despite the systematic improvement, the AIMD-based scheme cannot inherently reproduce zero-point-energy effects and related nuclear quantum delocalisation of the nuclei, especially the protons. (The only computational scheme that would have been capable of accounting for such effects would be based on the Path Integral Molecular Dynamics (PIMD).) Such delocalisation, according to the Heisenberg uncertainty principle, causes the protons to exhibit narrower NMDs (and thus, lower values of nuclear kinetic energies), compared to the values simulated in *ab initio* schemes treating nuclei as classical particles occupying single points in space^83^. An average proton NMD width of 4.78 Å$^{-1}$ corresponds to spatial delocalisation of ca. 0.209 Å. In the case of boron and oxygen, these values are 0.104 and 0.088 Å, respectively. Thus, protons in boric acid crystals explore regions of space around their local minima on the PES that are approximately twice as large as those explored by boron and oxygen nuclei, making the protons more likely to experience anharmonic regions of the PES. In consequence, the AIMD scheme is more likely to better compare with experimental values of NMD widths and nuclear kinetic energies, especially in the case of protons.

Apart from the isochoric specific heat, the neutron observable that is more sensitive to the low-to-mid energy vibrations than to the high-energy ones, and thus complements the NCS benchmarking of the *ab initio* simulations, is the incident neutron energy-dependent total neutron cross-section measured in the NT technique on VESUVIO. Qualitatively, the overall shape of the incident neutron energy dependency of the total scattering cross section is well approximated by all three types of  *ab initio* simulations: HLD, AIMD and AFGA, and the differences between the shapes of the total scattering cross section curves simulated using these three different simulation types are negligible, despite a noticeable blue shift in the high-energy part of the $^{1}$H VDoS (> 350 meV), which accompanies pronounced red shifts of the deformation modes contributing at about 100 and 150 meV), captured by the AIMD simulation (see Fig. 8b).

The leading contribution to the total scattering cross-section curve stems from the low and medium energy parts of the apVDoS of the hydrogen within single-phonon contribution to the multi-phonon expansion^84–86^. HLD and AIMD simulations yield similar shapes of the hydrogen apVDoS in this energy range, and thus the total neutron cross-section curves simulated using HLD and AIMD are very similar (see Fig. 8). A little more surprising is the very good agreement between the total cross sections obtained from the AFGA simulation and its HLD and AIMD counterparts. However, one has to bear in mind, that, in the case of the AFGA model, the boric acid, total scattering cross section is dominated by the contribution from the OH stretching band that is calculated as a weighted average over OH-stretch VDoSes taken from a large library of HLD simulations performed for a variety of organic compounds, in the spirit of machine learning^84^.

At epithermal incident neutron energies, the total scattering cross-section is no longer sensitive to differently thermally populated individual vibrational modes within atom-projected vibrational densities of states of individual isotopic species and starts depending on the epithermal scattering law, which in turn depends on the values of the total nuclear kinetic energies of individual isotopes^84,87^. It is worth noting that, in many theoretical studies, the epithermal part of the total scattering cross-section curves is modelled using the free-gas scattering law with a single value of the classical sample temperature used to model the motion of all isotopic species present in a system under investigation^84^. The values of the individual nuclear kinetic energies of hydrogen, boron, and oxygen in solid boric acid at room temperature obtained from the NCS experiments differ significantly from 38.7 meV, the value of the kinetic energy of nuclei in a Maxwell-Boltzmann gas of free, non-interacting particles at room temperature. The discrepancy is highest in the case of hydrogen (142 meV), followed by boron (53.5 meV), and oxygen (50.9 meV). As shown in the simulations in Section D in the Supplementary Information, this difference will manifest itself in the total cross-section of natural abundance boron already for incident neutrons of energies of ca 1 eV, at which the contribution to the total cross-section from neutron absorption in boron will have decreased to a level, at which the contribution form the scattering cross-section starts dominating. In the case of boron-11, where the contribution from the neutron absorption is negligible compared to boron-10, the value of the effective temperature in the scattering law at epithermal incident neutron energies will play an even bigger role.

The limits of detection and quantitation of boron and boric acid, determined using neutron Compton scattering, incident neutron energy-dependent transmission and *in situ* PGAA are much larger than the amounts of boron required to be detected in most BNCT protocols ^1^. Notwithstanding the inability to monitor in vivo boron uptake and no application to real-time dosimetry or biodistribution, these methods can be used in quality control of large batches of solid boron-carrier compounds and detection of certain high-energy materials, such as RDX@Nano-B composites^88^. Additionally, NCS can be employed to assess zero-point energy contributions to the stability of different polymorphs and isotopologues of boron-carrier compounds, and to investigate boron isotope fractionation in medical settings. Dose planning and biological-uptake studies must rely on much more sensitive approaches such as Inductively Coupled Plasma Mass Spectrometry or PGAA experiments performed after the neutron irradiation in controlled, well-shielded environments, outside the blockhouses of the neutron beamlines ^1^. Such settings, however, do not allow for concurrent detection of neutrons, as is the case with the VESUVIO beamline.

In summary, our thermal-to-epithermal neutron study of solid boric acid has demonstrated the potential of a multi-technique approach, augmented with global *ab initio* modelling of nuclear quantum effects and observables, to assess the feasibility of selected isotopic species for specific pharmaceutical applications and protocols relevant in nuclear medical settings. Our comparative analysis has revealed excellent agreement between first-principles predictions and experimental data, thereby validating the computational framework adopted in this study. The AIMD approach, operating under finite-temperature conditions, has proved particularly effective in capturing both structural and vibrational properties, including the full vibrational density of states as reflected in isochoric heat capacity, total neutron cross-section, and nuclear kinetic energies, as well as the INS response. Despite the inherent limitations of classical sampling, a degree of fortuitous error cancellation enables AIMD to reproduce experimental trends with remarkable fidelity. With the observed consistency extending to both structural and vibrational properties, the present model constitutes a robust reference for predicting VESUVIO neutron observables.

Applying such a global approach, one must be fully aware of the limitations and constraints of different techniques and protocols; a feasibility study like this one aims to address exactly that. As much as neutron Compton scattering and neutron transmission are methods of choice for the benchmarking of the *ab initio* computations that can yield valuable predictions of nuclear quantum observables, they cannot be easily used for real-time boron dosimetry or biodistribution. On the other hand, techniques such as prompt-$\gamma$ detection can be used for detecting isotopes in drugs and tissues at very low concentrations, but cannot be easily applied to basic studies of nuclear quantum dynamics. Thus, it is the power of the combined approach, concurrently applying all the above-mentioned methods, that yields the winning combination in the context of medical research and nuclear engineering. Notwithstanding this last remark, further upgrade of the VESUVIO thermal-to-epithermal neutron station can change things dramatically. Simple scaling up of the detection capabilities of epithermal neutrons by providing more detectors can bring the values of the LOD and LOQ of the NCS technique to levels characteristic of prompt-$\gamma$ detection. Without a doubt, further studies of this type are necessary to showcase the importance of broadband neutron detection in cancer research.

## Methods

### Ab initio simulations

Theoretical calculations for the triclinic phase of BA were performed under periodic boundary conditions (PBC) using the plane-wave pseudopotential framework as implemented in CASTEP version 21.11^89^. The initial unit cell was constructed based on the structural parameters for the $\hbox {H}_{3}$ $\hbox {BO}_{3}$-2A phase reported by Zachariasen et al.^35^ and subjected to electronic structure calculations. To this end, a set of hard norm-conserving pseudopotentials was generated on the fly and employed in combination with a plane-wave kinetic energy cutoff of 1400 eV. The choice of such a high cutoff was dictated by the need to ensure robust convergence of total energies and interatomic forces, particularly in systems containing light elements like hydrogen, where rapidly varying wavefunctions demand higher spatial resolution^90^. This allows for the minimisation of basis set incompleteness effects and ensures the numerical stability of all structural and vibrational predictions. To improve numerical accuracy while maintaining computational efficiency, integration over the Fast Fourier Transform (FFT) grid in real space was employed, utilising a double-grid technique to evaluate charge density and exchange–correlation contributions. A standard grid scaling factor of 2.0 and a finer grid scaling of 3.0 were used to control the resolution of the FFT grid. In this approach, the smoother electronic wavefunctions were evaluated on the standard grid, whereas the more localised charge density was computed on the finer grid, allowing for better spatial resolution of rapidly varying quantities near the atomic cores. Spin-polarisation and spin–orbit coupling effects were neglected due to the non-magnetic nature of the system.

The exchange–correlation energy was defined using the Perdew–Burke–Ernzerhof (PBE) functional, combined with the semi-empirical pairwise van der Waals corrections by Tkatchenko and Scheffler (TS), hereafter referred to as PBE-TS.^91,92^ The Monkhorst-Pack (MP) grid was adjusted to maintain a constant spacing in reciprocal space of 0.05 Å$^{-1}$, and the self-consistent-field (SCF) energy was converged with a tolerance of 1 $\times$ 10$^{-8}$ eV per atom throughout this work. The Pulay mixing scheme with a fixed charge occupancy was employed to accelerate convergence of the SCF cycles. For the geometry optimisation, the Hellmann–Feynman forces acting on individual ions were minimised to 1 $\times$ 10$^{-3}$ eV/Å, and the associated stress components were converged to within 10$^{-4}$ GPa at ambient pressure. The full cell relaxation was performed using the limited-memory Broyden–Fletcher–Goldfarb–Shanno (LBFGS) algorithm, employing an exponential preconditioner and a modulus estimate of 500 GPa. The finite basis set corrections were included using three sampling points. The equilibrium structures were subjected to vibrational analysis and the finite-temperature BOMD simulations.

The harmonic phonon calculations across the first Brillouin zone of the $\hbox {H}_{3}$ $\hbox {BO}_{3}$-2A structure were carried out at 0 K using linear-response theory, as implemented in CASTEP^33^. This approach enables the direct evaluation of the analytical phonon energies and their corresponding eigenvectors, which serve as input for evaluating the neutron observables of interest in the present work. The phonon band structures were obtained by interpolating the dynamical matrix across the $\hbox {1}^{\hbox {st}}$ Brillouin zone, based on force constants calculated via DFPT. From the interpolated phonon dispersion, the total and atom-projected VDoSes were evaluated, enabling clear assignment of vibrational contributions from each atomic sublattice (e.g., B, O, and H) to specific phonon modes. The phonon eigenvalues and eigenvectors served as the input for modelling the vibrational and nuclear quantum observables, which were performed according to the protocol previously described in our work on illicit substance detection using VESUVIO spectrometer^48^. The analysis of the vibrational modes was performed utilising the PDIelec code ^93^ and the INS simulations were performed with the help of oCLIMAX.^94^.

In order to investigate mechanical anharmonicity and thermally induced spectral broadening, the first-principles MD simulations were conducted at both low (10 K) and ambient (300 K) temperature conditions. The DFT-MD simulations were performed under identical numerical settings to those used in the harmonic calculations, yet employing a larger 2$\times$2$\times$2 supercell to minimise the finite-size effects. The equations of motion were integrated using a time step of 0.5 fs, ensuring accurate resolution of atomic vibrations. The MD simulations were initiated in the constant-pressure, constant-temperature (NPT) ensemble to allow full equilibration of volume and temperature. This was followed by simulations in the canonical (NVT) ensemble to stabilise thermal fluctuations, and ultimately concluded within the microcanonical (NVE) ensemble to preserve the total energy. NPT simulations were carried out for 10000 steps, with temperature regulated using a Nosé–Hoover thermostat and a damping constant of 5.0 fs. Pressure control was implemented via an Andersen–Hoover barostat, which allowed for isotropic volume fluctuations while constraining the simulation cell shape, probed using a damping constant of 1.0 ps. Ionic positions were updated using a first-order extrapolation scheme with on-the-fly fitting, ensuring stable and predictive integration. Stress tensors were evaluated at each MD step to accurately determine the elastic response. From the obtained NPT trajectory, an averaged supercell was constructed over the simulation frames and used in the subsequent NVT AIMD simulation with the same integration step. An extended Lagrangian Born–Oppenheimer MD scheme (XL-BOMD) was enabled to propagate atomic positions and electronic wavefunctions. The NVT ensemble was maintained at target temperatures (10 K and 300 K) for 10000 steps, utilising a Nosé–Hoover thermostat with an ionic damping constant of 5.0 fs. The stress-tensor calculations were disabled. At the final stage, the system was decoupled from the Nose-Hoover thermostat, and the run was continued for 50000 steps in a microcanonical ensemble using the same numerical settings. The simulations of the INS spectrum for a direct comparison with TOSCA data incorporated a numerical evaluation of the Debye–Waller tensor, accounting for thermal atomic displacements through trajectory-based averaging according to the oCLIMAX implementation^94^. The analysis of the NVE trajectory to extract atom-projected VDoSes was conducted using the TRAVIS package^95^.

Both the HLD and AIMD computational routes were employed to predict the values of nuclear kinetic energies and the widths of nuclear momentum distributions from atom-projected VDoSes (apVDoSes) and the heat capacity at constant volume from total VDoSes. In the harmonic approximation, the atom-projected VDoSes, $G_{M,HLD}(\omega )$, were obtained from the dynamical matrix using the DFPT framework using^29^:

3 $$\begin{aligned} G_{M,HLD}(\omega ) = \frac{1}{3 N_q} \sum _{q\in 1BZ} \sum _{\lambda =1}^{N_{\lambda }} e_M(\lambda ,q)^2 \delta (\omega -\omega (\lambda ,q)) \end{aligned}$$

where $\omega (\lambda ,q)$ are phonon frequencies and $e_M(\lambda ,q)$ are the magnitudes of the phonon eigenvectors for atoms $\textit{M}$, the summation runs over all *q* vectors contained in the first Brillouin zone, $N_q$, and all phonon branches (q-dependent modes) $\lambda$, and $G_{TOT,HLD}(\omega ) = \sum _{M} G_{M,HLD}(\omega )$.

In order to capture temperature-induced softening or hardening of phonon modes and/or broadening of phonon bands, the total (atom-projected) VDoSes ($G_{TOT,AIMD}(\omega , T)$ and $G_{M,AIMD}(\omega , T)$, respectively) were also computed from trajectories obtained in NVE AIMD simulations. The $G_{M,AIMD}(\omega , T)$ for a particular atom *M* was calculated as the Fourier transform of its Velocity Auto Correlation Function (VACF) with no corrections for the frequency shift associated with the Verlet integrator.^28,29^:

4 $$\begin{aligned} G_{M,AIMD}(\omega , T)=\int _{0}^{\infty }\frac{<\nu (t)_{M}\nu (0)_{M}>}{<\nu (0)_{M}^{2}>}exp\left( -i\omega t \right) dt, \end{aligned}$$

where $\nu (t)_{M}$ is the velocity magnitude of atom $\textit{M}$ at time $\textit{t}$ and $G_{TOT,AIMD}(\omega , T) = \sum _{M} G_{M,AIMD}(\omega , T)$. We note here the implicit temperature dependence of $G_{TOT,AIMD}(\omega , T)$ and $G_{M,AIMD}(\omega , T)$ as they stem from a series of AIMD simulations performed subsequently in NPT, NVT and NVE ensembles. In the NPT and NVE calculations, the temperature was stabilised by means of a thermostat as described above, and the NVE simulation was performed as a continuation of the NVT simulation. The inspection of temperature fluctuations along the NVE trajectory revealed that, despite the lack of thermostatting, the temperature remained stable, fluctuating around the average value of 298 K with a standard deviation of only 11 K. Let us bear in mind, however, that the AIMD simulations described above yield semi-classical representations of the vibrational density of states. All vibrational modes present in $G_{TOT,AIMD}(\omega , T)$ and $G_{M,AIMD}(\omega , T)$ contribute equally to the total vibrational energy, and the contribution of motion of frequency $\omega$ is proportional to the temperature but independent of the frequency of the vibration^96^. However, a quantum character can be introduced into $G_{TOT,AIMD}(\omega , T)$ and $G_{M,AIMD}(\omega , T)$ by multiplying them by the Bose-Einstein occupation factor, $\coth \left( \frac{\omega }{2k_BT} \right)$, which represents the frequency-dependent population of vibrational levels of the quantum harmonic oscillator at a given temperature ^96^. Thus, one can express the nuclear kinetic energy and heat capacity at constant volume using effectively the same formulas, differing only by the origin of the total (atom-projected) VDoSes. There is, however, one important distinction. In the HLD computational route, the total (atom-projected) VDoSes are calculated at 0 K and then extrapolated to the entire temperature region of interest. In the case of the AIMD route, however, one has to compute $G_{TOT,AIMD}(\omega , T)$ and $G_{M,AIMD}(\omega , T)$ at any given temperature separately (from a separate series of AIMD simulations in the NPT, NVT, and NVE ensembles).

The nuclear kinetic energies, $E_{k,M}$, were obtained using the relation^29^:

5 $$\begin{aligned} E_{k,M,(HLD,AIMD)} (T) = \frac{3\hbar }{4} \int \omega ~G_{M,(HLD,AIMD)}(\omega )~\coth \left( \frac{\omega }{2k_BT} \right) d\omega , \end{aligned}$$

It is worth noting that Eq. 5 is strictly valid in the HLD approach as it takes into account the result of the application of the virial theorem to the theory of quantum harmonic oscillator. Namely, it uses the fact that the expectation value of the kinetic vibrational energy is half the expectation value of the total vibrational energy^43,97^. However, for a wide class of realistic anharmonic potential energy surfaces, the anharmonic version of the virial theorem produces ratios of kinetic to total vibrational energy expectation values that are very similar to the harmonic counterpart, and hence the AIMD counterpart of Eq. 5 is justified.

The spherically averaged values of the widths of Gaussian nuclear momentum distributions, $\sigma _{M,(HLD,AIMD)}^2$, were then obtained from Eq. 5 using^29^:

6 $$\begin{aligned} E_{k,M,(HLD,AIMD)} = \frac{3\hbar ^2\sigma _{M,(HLD,AIMD)}^2}{2M} \end{aligned}$$

The heat capacity at constant volume, calculated within the HLD route, $C_{V,(HLD)}(T)$, was evaluated as a partial derivative of the total vibrational energy over temperature using $G_{TOT,HLD}(\omega , T)$  ^96^:

7 $$\begin{aligned} C_{V,HLD}(T) = \int _{0}^{\infty } k_B \left( \frac{\hbar \omega }{k_B T} \right) ^2 \frac{e^{\hbar \omega / k_B T}}{\left( e^{\hbar \omega / k_B T} - 1\right) ^2} G_{TOT,HLD}(\omega ) \, d\omega , \end{aligned}$$

Importantly, within the harmonic approximation, $C_{V,HLD}$ is proportional to the partial derivative of the total kinetic energy of the system ^96^, $C_{V,HLD} = 2\frac{\partial E_{k,HLD}}{\partial T}$.

The calculation of $C_{V,AIMD}(T)$ is also possible. However, it is worth noting that the partial derivative of the total vibrational energy with respect to temperature, computed within the AIMD scheme, can be obtained using two computational routes. In the first route, if $G_{TOT, AIMD}(\omega , T)$ is almost constant within the temperature range of interest, one can approximate it by its form computed at a specific temperature, $G_{TOT, AIMD}(\omega , T=T_0)$, and differentiate only the Bose-Einsten population factor over temperature, which leads to an expression that is analogous to Eq. 7 ^96^:

8 $$\begin{aligned} C_{V,AIMD}(T) = \int _{0}^{\infty } k_B \left( \frac{\hbar \omega }{k_B T} \right) ^2 \frac{e^{\hbar \omega / k_B T}}{\left( e^{\hbar \omega / k_B T} - 1\right) ^2} G_{TOT,AIMD}(\omega , T=T_0) \, d\omega , \end{aligned}$$

In the second case, for a grid of temperature points, $T_i$, separate AIMD simulations need to be first performed to obtain individual $G_{TOT, AIMD}(\omega , T_i)$, and the product of $G_{TOT, AIMD}(\omega , T_i)$ and the Bose-Einstein population factor needs to be numerically differentiated on this grid and then integrated over the $\omega$ domain. Moreover, in light of what has been said above, the relation $C_{V,(AIMD)} = 2\frac{\partial E_{k,(AIMD)}}{\partial T}$ can also be used for a relatively wide class of anharmonic potentials sampled in the AIMD scheme.

The heat capacity at constant pressure, $C_P$, directly accessible in experiments, can be derived from $C_V$ using the isotropic thermal expansion coefficient $\alpha$ and the isotropic compressibility modulus $\beta$ using^96^:

9 $$\begin{aligned} C_P(T) = C_V(T) + T~V_0~\frac{\alpha ^2}{\beta } \end{aligned}$$

where $V_0$ is the molar volume. In practice, under normal conditions, in solids, the difference between $C_P$ and $C_V$ is of the order of a few per cent^98^.

The influence of the boron isotope effects on the shapes of the simulated INS spectra, the values of the NMD widths and nuclear kinetic energies, and the shapes of the total neutron cross-sections recorded as functions of incident neutron energies was examined by calculating these observables using apVDoSes simulated within the HLD approach for different boron isotope masses. The apVDoSes were obtained by re-diagonalising the dynamic matrices calculated for different boron isotopes without rerunning the electronic structure calculation, using the PHONONS functionality in CASTEP^33^.

### Sample preparation and sample environment

Boric acid powder of 99% purity was purchased from Sigma Aldrich (product number B0394) and used directly in the NCS and NT experiments. To determine the values of LOD and LOQ, different amounts of boric acid powder were loaded into flat aluminium containers positioned perpendicular to the incoming neutron beam. These containers had cross-sectional areas matching the diameter of the neutron beam on VESUVIO, and their interiors were filled with flat aluminium disks to match the container volume. Five samples containing 0.290, 0.575, 1.152, 1.580, and 2.299 g of boric acid were prepared by carefully weighing the as-obtained powder. The accuracy of the weight determination was 0.001 g in each case. The samples were measured at 300 K, with the temperature values stabilised using a closed-circuit refrigerator (CCR) mounted inside the VESUVIO beamline. For each sample, data were collected for a period of time corresponding to an integrated proton current within the ISIS synchrotron of 1600 µAh.

### Prompt gamma activation analysis

The PGAA spectra were measured using a high-purity germanium (HPGe) N-type $\gamma$ detector (model GMX50P4-83-CW), red out by the Ortec amplifier (model 257N) and multichannel analyser (model DSPEC-50). The used front-end electronics were set in a way to simulate the energy resolution of a high-density scintillating material like NaI(Tl), YAG:Ce or $\hbox {LaBr}_3$: Ce,Sr, which were demonstrated to be a good solution for BNCT dose monitoring in clinical conditions^54^. The detector was calibrated before each sample irradiation using standard elemental $\gamma$-ray sources: $^{22}$Na (511 and 1275 keV), $^{137}$Cs (661.7 keV) and $^{60}$Co (1173.2 and 1332.5 keV). The $\gamma$ spectra of five samples containing 0.290, 0.575, 1.152, 1.580, and 2.299 g of solid boric acid in powder form and an empty aluminium container (background) were recorded concurrently with the neutron data acquisition on VESUVIO. The measurements were performed at 300K, and the temperature was stabilised using the ORTEC Integrated Cryocooling System (model CFG-ICS-P4). For each boric acid sample, the recorded raw spectra were corrected by background subtraction and normalised to the measurement duration and the integrated proton current of the ISIS accelerator, corresponding to the incident neutron flux. To determine the number of recorded $\gamma$ quanta originating from the neutron capture on boron nuclei, the spectra were fitted in the $\gamma$ energy range of 400 – 550 keV. In this energy range, the spectra consisted of two overlapping peaks, a peak at 478 keV due to neutron capture on boron and a peak due to the annihilation photons at 511 keV, which originates from pair production of high-energy $\gamma$ rays (such as the 2.2 MeV $\gamma$ rays generated by hydrogen neutron capture reactions)^54^. An additional difficulty in fitting the spectra recorded in the energy range of 400 – 550 keV was attributed to the fact that the 478 keV $\gamma$ ray is emitted in motion by the $^{7}$Li nuclei, causing Doppler broadening of the peak at 478 keV^54^. Also, in the case of the VESUVIO beamline, similarly to other accelerator-driven neutron sources, background signals at the exact energy of 478 keV are additionally generated by boron neutron capture reactions occurring outside of the neutron target, in the blockhouse walls, and in various materials present in the blockhouse ^54^. For these reasons, the peaks present in the energy range of 400 – 550 keV were deconvoluted by fitting either two Gauss or two Lorentz functions. The values of LOD and LOQ were then calculated using the linear dependence of the integral intensities of the fitted peaks at 478 keV on the boron mass in samples of boric acid of different masses.

### Neutron Compton scattering

A detailed description of the raw NCS data treatment is described elsewhere^29,41–48^. Briefly, in the case of boric acid, NCS spectra, recorded in the Time-Of-Flight (TOF) domain, *t*, are superpositions of recoil peaks of hydrogen, boron and oxygen, as well as aluminium from the sample container and the CCR. The contributions from the CCR are subtracted by measuring separate spectra for an empty CCR. The NMDs underlying the recoil peaks are modelled as Gaussian momentum distributions, $J_M(y_M(t))$, when plotted in the longitudinal momentum spaces of individual masses $y_M(t)$:

10 $$\begin{aligned} J_M(y_M(t))= \frac{1}{\sqrt{2\pi \sigma _M^2}}\exp {\left( -\frac{y_M(t)^{2}}{2\sigma _M^2}\right) } \end{aligned}$$

In fitting the NCS spectra, the ratios of the relative integral intensities, $\frac{I_M}{I_M';}$ of the recoil peaks of isotopic species of masses *M* and $M'$ are linearly constrained^47,48,51,99–101^:

11 $$\begin{aligned} \frac{I_M}{I_M'} = \frac{4\pi b_M^2 c_M}{4\pi b_M'^2 c_M'} \end{aligned}$$

These constraints were applied for peaks of hydrogen, boron and oxygen, but not for the aluminium peak, because the real number of aluminium atoms per formula unit of the boric acid sample is not known a priori.

The raw NCS data were corrected for multiple-scattering and $\gamma$ background effects using standard procedures implemented in the Mantid computational environment^29,41–48^.

### Neutron transmission

Neutron transmission data were processed employing an established protocol described elsewhere^48,84,87,102–104^. The incident neutron energy, $E_0$, dependent sample transmission was defined as:

12 $$\begin{aligned} T_s(E_0) = \exp {\left( -S_s(E_0)\right) } \end{aligned}$$

where $S(E_0)$ is the $E_0$-dependent sample scattering power.

Moreover, the total transmission of the sample and container was expressed as $T_S (E_0) T_c(E_0)$, and the transmission of the empty container, $T_c(E_0)$, was measured in a separate experiment.

The plateaus present in the $E_0$-dependent transmission curves of samples for $10eV< E_0 < 100eV$, defined as $S_{10eV-100eV} = 1 - T_{10eV-100eV}$, were expressed as:

13 $$\begin{aligned} S_{10eV-100eV} = d n \sum _{i=1}^{N} c_i \frac{4\pi b_i^2}{(1 + m/M_i)^2} \end{aligned}$$

where *d* is the sample thickness along the direction of the incident neutron beam, *n* is the sample number density, $c_i$ is the number of the i-th isotopic species per sample formula unit, $4\pi b_i^2$ is the value of the total bound neutron scattering cross-section (with $b_i^2$ being the value of the total bound scattering length) of the i-th isotopic species, *m* and $M_i$ are the neutron and isotopic species masses, both expressed in atomic mass units, and *N* is the total number of different types of isotopic species per sample formula unit. In the absence of the exact values of *d* and *n*, the total experimental neutron cross-section curve was normalised for $10eV< E < 100eV$ to the value of $\sum _{i=1}^{N} c_i \frac{4\pi b_i^2}{(1 + m/M_i)^2}$.

Modelling of the total neutron cross-section recorded as a function of incident neutron energy was performed with the NCRYSTAL computational environment^105^. The total cross-section was modelled as a sum of isotope-dependent scattering and absorption cross-sections. In the simulated total neutron cross-section curves, nuclear resonances were not considered as they are not observed from H, B and O for incident neutron energy values between 0.1 meV and 100 eV.

For the specific case of the solid boric acid, due to the relatively large number density of incoherently scattering hydrogen in the sample, the modelling of the total scattering cross-section was performed within the incoherent approximation. The multi-phonon expansion method was employed using apVDoSes obtained from the HLD, AIMD and AFGA approximations. In the case of the AFGA approximation, the apVDoSes were modelled as linear combinations of the hydroxyl (OH) group vibrational densities of states and VDoSes obtained from the Debye approximation for boron.

It is worth noting that, in the epithermal incident neutron energy limit, the total scattering cross-section simulated using the multi-phonon expansion method converges to a result given by the epithermal scattering law, $\sum _{i=1}^{N} c_i \frac{4\pi b_i^2}{(1 + m/M_i)^2}$ ^84^. This result can be obtained by keeping $E_0$ fixed while performing double numerical integration over the neutron energy transfer, $\omega = E_1 - E_0$ (where $E_1$ is the final neutron energy) and solid angle, $d\Omega = 2\pi \sin {\theta } d\theta$ (where $\theta$ is the neutron scattering angle) of the double-differential scattering cross-section, $\frac{d^2\sigma }{d\omega d\Omega }$ ^84^:

14 $$\begin{aligned} \frac{d^2\sigma }{d\omega d\Omega } = \sqrt{\frac{E1}{E0}}~\sum _{i=1}^{N} c_i \frac{4\pi b_i^2}{(1 + m/M_i)^2}~\frac{\exp (-\frac{(\omega -\omega _{r,i})^2}{6\omega _{,i}rE_{k,i}})}{\sqrt{6\pi \omega _{r,i}E_{k,i}}} \end{aligned}$$

where $\omega _{r,i} = \frac{\hbar ^2 q^2}{2M_i}$ is the recoil energy of a nucleus of mass $M_i$, $E_{k,i}$ is the nuclear kinetic energy of a nucleus of mass $M_i$, and *q* is the neutron momentum transfer, $q^2 = 2mE_0/\hbar ^2 + 2mE_1/\hbar ^2 - 2m\sqrt{E_0E_1}/\hbar ^2 \cos {\theta }$.

The absorption cross section, $\sigma _{abs,M}(E_0)$, for each isotopic species of mass $M_i$ was included as:

15 $$\begin{aligned} \sigma _{abs,M}(E_0) = \sigma _{abs,M}(E_{ref})\sqrt{\frac{E_{ref}}{E_0}} \end{aligned}$$

where $\sigma _{abs,M}(E_{ref})$ is the tabulated value of the neutron absorption cross-section of a given isotopic species measured at the thermal incident neutron energy $E_{ref}$ of 25.3 meV^106^.

## Supplementary Information

Supplementary Information.

## Data Availability

The datasets used and/or analysed during the current study are available from the corresponding author upon reasonable request.
