# Supplementary material for: Nuclear quantum dynamics of boric acid as probed by a thermal-to-epithermal neutron station
Source: Sci Rep. 2025 Nov 27;15:45447. doi: 10.1038/s41598-025-29342-2 (PMC12749873; doi:10.1038/s41598-025-29342-2)
Supplement: Supplementary file 1 — Supplementary Information. [file 41598_2025_29342_MOESM1_ESM.pdf]

Nuclear quantum dynamics of boric acid as probed by a thermal-to-epithermal neutron station

Katarzyna Dziedzic-Kocurek[1,+], **Michał Silarski**[1,+], **Kacper Drużbicki**[2,+], **Patryk Grabowski**[1], **and Matthew Krzystyniak**[3,*,+]

[1]Faculty of Physics, Astronomy and Applied Computer Science, M. Smoluchowski Institute of Physics, Department of Experimental Particle Physics and Applications, Jagiellonian University, 30-348 Kraków, Poland
[2]Centre of Molecular and Macromolecular Studies, Polish Academy of Sciences, Sienkiewicza 112, 90-363 Lodz, Poland
[3]ISIS Neutron and Muon Source, STFC Rutherford Appleton Laboratory, OX11 0QX, United Kingdom
[*]matthew.krzystyniak@stfc.ac.uk
[+]these authors contributed equally to this work

Contents

S1 Molecular structure from *ab initio* modelling 2

S2 Analysis of the zone-centre phonon modes 3

S3 Phonon properties beyond the Γ-point 15

A Calculation of the isotope effect on the widths of the nuclear momentum distributions and the zero-point vibrational energies of boron 16

B Isotope effects in the atom-projected VDoS spectra of boric acid 17

C Isotope effects in the INS spectra of boric acid 18

D Isotope effects in total cross-section of boric acid 19

References 19

S1 Molecular structure from *ab initio* modelling

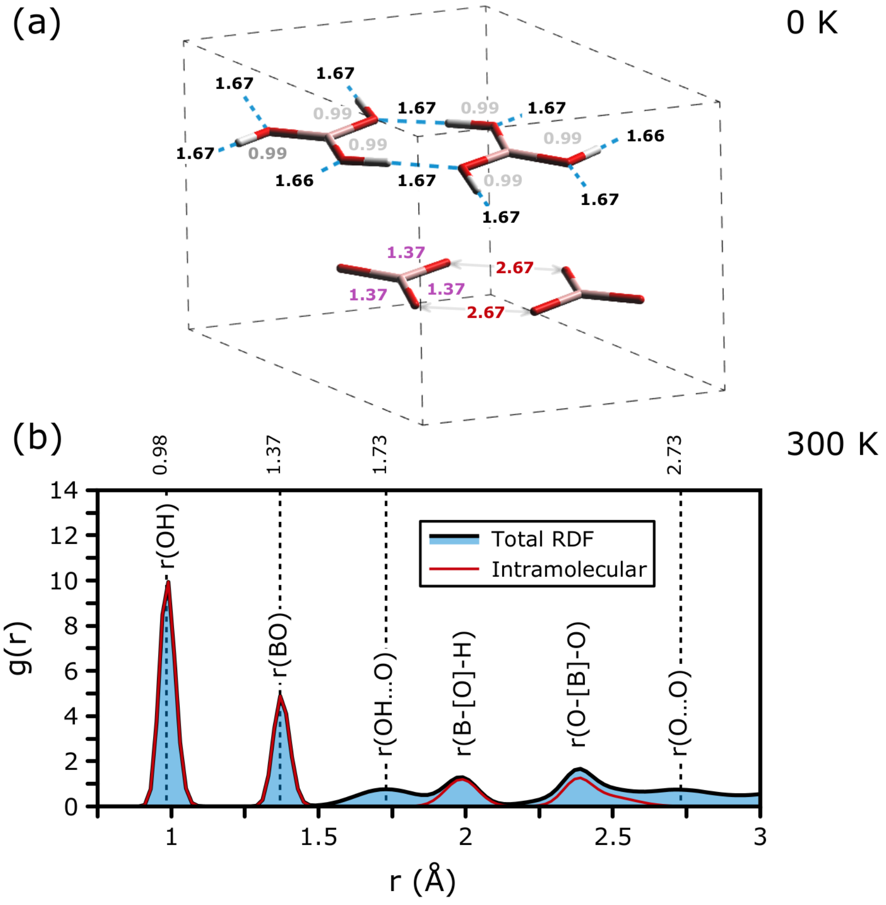

Figure S1. (a) Interatomic distances within and between boric acid molecules in the optimised 0 K structure of the triclinic (*P*-1) phase, obtained using the PBE-TS exchange-correlation functional. (b) Radial distribution functions (RDFs) for atomic pair correlations computed from a 25 ps *ab initio* NVE MD trajectory at 300 K. The total RDF (blue) is decomposed into intramolecular (red) contributions. Key peaks correspond to r(O–H), r(B–O), r(O···H), and r(O···O), allowing clear identification of intra- vs. intermolecular structure.

Table S1. Comparison of the bond lengths and the intermolecular contacts (in Å) from neutron diffraction on a perdeuterated $D_3{}^{11}BO_3$ specimen (300 K) [Craven, B. M. & Sabine, T. M. *Acta Crystallogr.* 20, 214—219 (1966).] and the results of PBE-TS calculations at 0 K (DFT) and 300 K (DFT-MD).

Bond Type	NPD 300 K	DFT 0 K	DFT-MD 300 K
**O–H bond lengths**			
Mean O–H	0.972	0.992	0.988
Range	0.946–1.004	0.992–0.993	0.958–1.018
**O···H hydrogen bond lengths**			
Mean O···H	1.744	1.670	1.754
Range	1.715–1.780	1.662–1.675	1.617–1.891
**B–O bond lengths**			
Mean B–O	1.370	1.371	1.376
Range	1.346–1.387	1.370–1.373	1.339–1.412
**O···O intermolecular contacts (Mol1...Mol2)**			
O···O	2.727	2.664	2.686
O···O	2.724	2.666	2.779
**B···B distances (Mol1...Mol2)**			
B···B	3.642	3.562	3.620

S2 Analysis of the zone-centre phonon modes

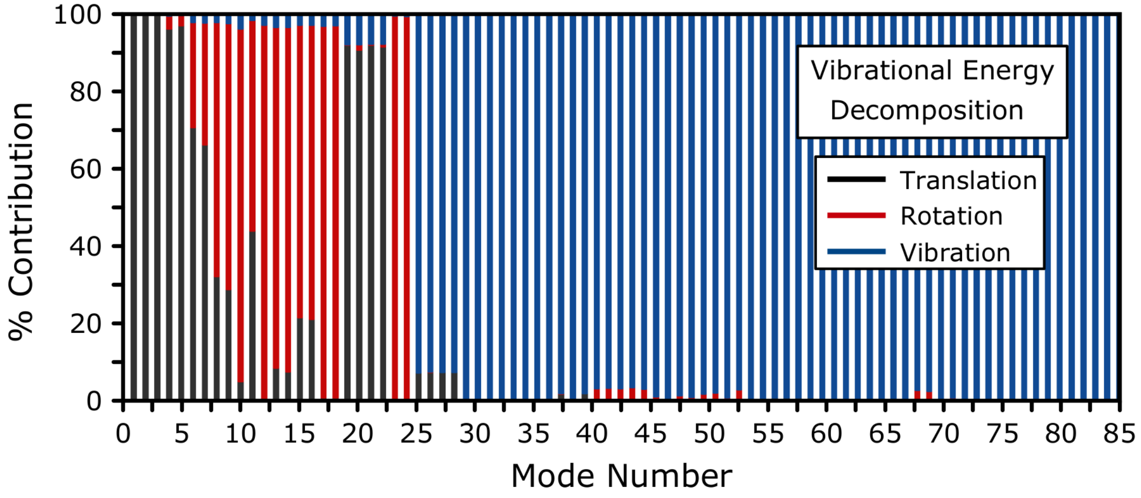

Figure S2. Vibrational energy decomposition of the zone-centre modes for the triclinic (*P*-1) phase of boric acid, obtained from harmonic lattice dynamics (HLD) calculations using density-functional perturbation theory (DFPT) with the PBE-TS functional. External modes are further resolved into translational (black) and rotational (red) contributions of the molecular centroids. Internal vibrational contributions are shown in blue.

Table S2. Vibrational mode energies (in meV), symmetry classification (A_u or A_g), and assigned mode character for all 84 zone-centre normal modes of the triclinic (*P*-1) phase of boric acid. Mode energies were obtained using Γ-point density-functional perturbation theory (DFPT) within the PBE-TS exchange–correlation functional, in the framework of harmonic lattice dynamics (HLD).

Mode	*E* (meV)	Sym	Assignment	Mode	*E* (meV)	Sym	Assignment	Mode	*E* (meV)	Sym	Assignment
1	0.0	Au	Acoustic	29	69.4	Au	δ(O-B-O)	57	149.7	Ag	δ(B-O-H) + ν(B-O)
2	0.0	Au	Acoustic	30	69.4	Ag	δ(O-B-O)	58	149.9	Au	δ(B-O-H) + ν(B-O)
3	0.0	Au	Acoustic	31	69.8	Au	δ(O-B-O)	59	153.5	Au	δ(B-O-H) + ν(B-O)
4	4.4	Ag	Shearing	32	69.8	Ag	δ(O-B-O)	60	153.6	Ag	δ(B-O-H) + ν(B-O)
5	5.2	Ag	Shearing	33	76.9	Au	$\gamma(BO_3)$	61	153.8	Ag	δ(B-O-H) + ν(B-O)
6	10.3	Ag	$\tau(BO_3)$	34	77.4	Ag	$\gamma(BO_3)$	62	154.1	Au	δ(B-O-H) + ν(B-O)
7	11.3	Au	$\tau(BO_3)$	35	79.9	Au	$\gamma(BO_3)$	63	172.1	Au	γ(B-O-H) + ν(B-O)
8	12.2	Ag	$\tau(BO_3)$	36	80.5	Ag	$\gamma(BO_3)$	64	172.7	Au	γ(B-O-H) + ν(B-O)
9	13.3	Ag	$\tau(BO_3)$	37	95.7	Au	γ(B-O-H)	65	173.0	Ag	δ(B-O-H) + ν(B-O)
10	15.0	Au	$\tau(BO_3)$	38	95.9	Au	γ(B-O-H)	66	173.8	Au	δ(B-O-H) + ν(B-O)
11	16.0	Ag	$\tau(BO_3)$	39	98.5	Ag	γ(B-O-H)	67	175.9	Ag	γ(B-O-H) + ν(B-O)
12	16.4	Au	$\tau(BO_3)$	40	100.2	Ag	γ(B-O-H)	68	176.9	Au	γ(B-O-H) + ν(B-O)
13	17.6	Ag	$\tau(BO_3)$	41	102.5	Ag	γ(B-O-H)	69	180.0	Au	γ(B-O-H) + ν(B-O)
14	17.8	Au	$\tau(BO_3)$	42	102.9	Au	γ(B-O-H)	70	181.2	Ag	γ(B-O-H) + ν(B-O)
15	18.4	Au	$\tau(BO_3)$	43	105.1	Au	γ(B-O-H)	71	181.7	Ag	γ(B-O-H) + ν(B-O)
16	19.6	Ag	$\tau(BO_3)$	44	107.0	Au	γ(B-O-H)	72	181.8	Ag	γ(B-O-H) + ν(B-O)
17	25.9	Ag	ν(O...O)	45	107.4	Au	γ(B-O-H) + $\nu_{sym.}(BO_3)$	73	379.7	Ag	$\nu_{asym.}$(O–H)
18	25.9	Au	ν(O...O)	46	107.4	Ag	γ(B-O-H) + $\nu_{sym.}(BO_3)$	74	379.8	Au	$\nu_{asym.}$(O–H)
19	29.6	Ag	ν(O...O)	47	107.6	Au	γ(B-O-H) + $\nu_{sym.}(BO_3)$	75	384.6	Au	$\nu_{asym.}$(O–H)
20	30.2	Au	ν(O...O)	48	107.6	Ag	γ(B-O-H) + $\nu_{sym.}(BO_3)$	76	385.1	Ag	$\nu_{asym.}$(O–H)
21	30.2	Ag	ν(O...O)	49	108.5	Au	γ(B-O-H) + $\nu_{sym.}(BO_3)$	77	386.1	Ag	$\nu_{asym.}$(O–H)
22	30.4	Au	ν(O...O)	50	108.9	Ag	γ(B-O-H)	78	386.4	Au	$\nu_{asym.}$(O–H)
23	46.7	Ag	ν(O...O)	51	109.5	Ag	γ(B-O-H)	79	394.8	Au	$\nu_{asym.}$(O–H)
24	47.3	Au	ν(O...O)	52	110.4	Ag	γ(B-O-H)	80	395.1	Au	$\nu_{asym.}$(O–H)
25	62.5	Ag	δ(O-B-O)	53	144.6	Au	δ(B-O-H) + ν(B-O)	81	395.8	Ag	$\nu_{asym.}$(O–H)
26	62.6	Au	δ(O-B-O)	54	144.7	Ag	δ(B-O-H) + ν(B-O)	82	396.3	Au	$\nu_{asym.}$(O–H)
27	62.8	Au	δ(O-B-O)	55	149.4	Ag	δ(B-O-H) + ν(B-O)	83	410.2	Ag	$\nu_{sym.}$(O–H)
28	62.8	Ag	δ(O-B-O)	56	149.5	Au	δ(B-O-H) + ν(B-O)	84	412.6	Au	$\nu_{sym.}$(O–H)

Legend: ν = stretching, δ = in-plane bending (scissoring/rocking), γ = out-of-plane wagging, τ = librational (twisting) motion.

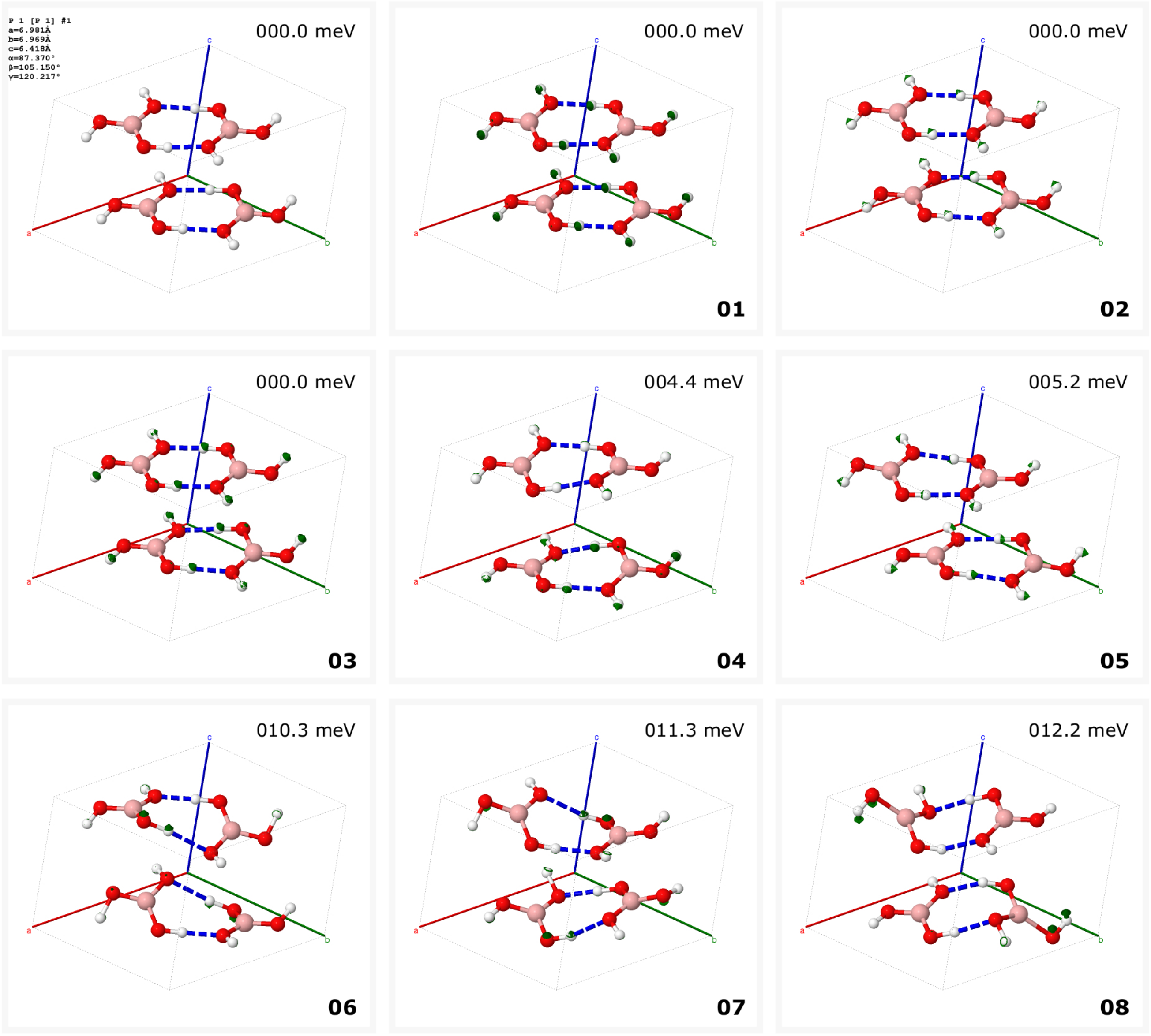

Figure S3. Zone-centre vibrational eigenmode displacement patterns for triclinic (*P*-1) boric acid (modes No. 1–8), computed via harmonic lattice dynamics (HLD) using DFPT and the PBE-TS functional. The atomic displacements are visualised as green vectors. Atoms are represented as solid spheres: hydrogen (green), boron (pink), and oxygen (red). Mode indices correspond to those listed in Table S1. The inset in the left panel shows the equilibrium (undisplaced) molecular structure for reference.

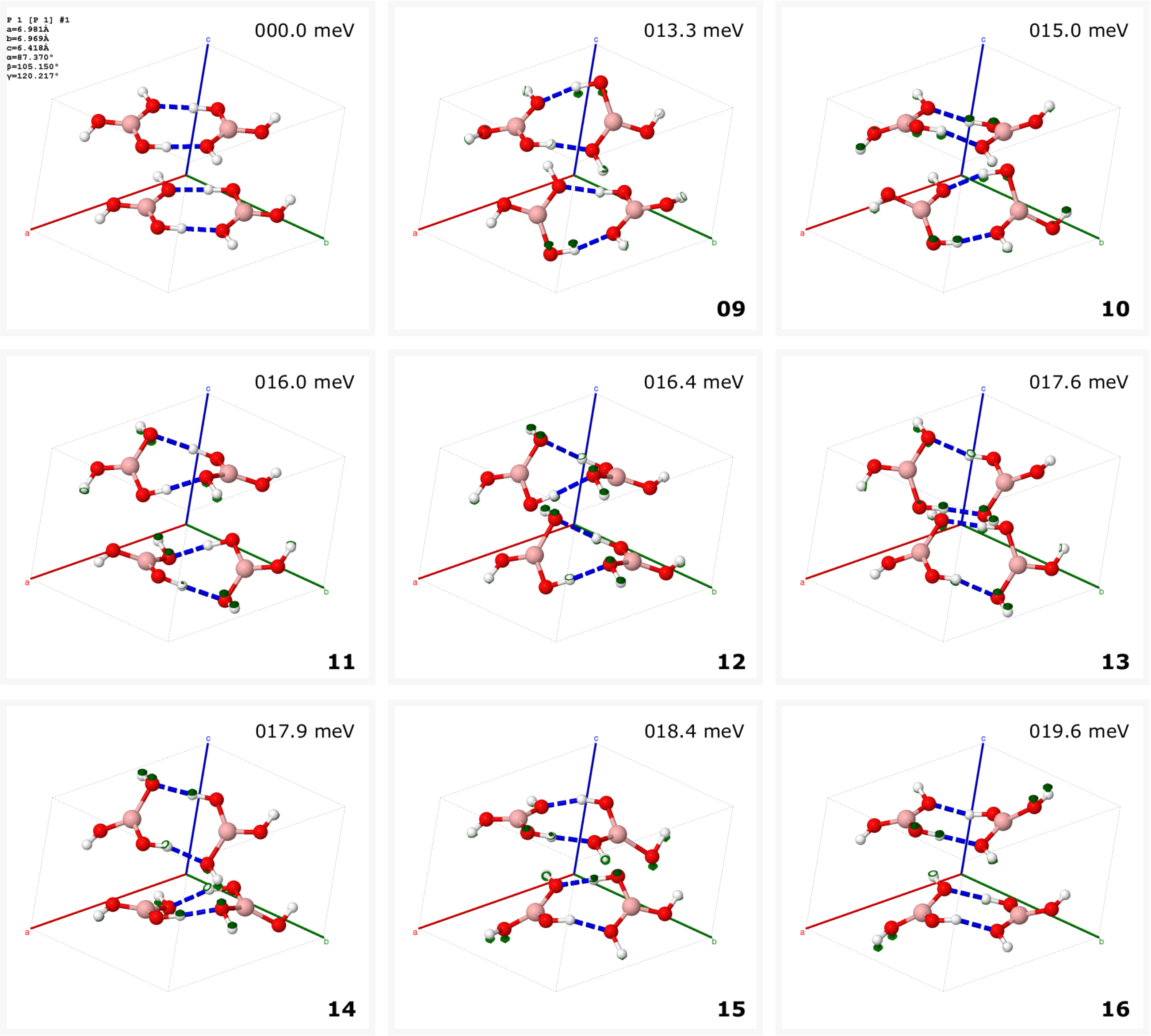

Figure S4. Zone-centre vibrational eigenmode displacement patterns for triclinic (*P*-1) boric acid (modes No. 9–16), computed via harmonic lattice dynamics (HLD) using DFPT and the PBE-TS functional. The atomic displacements are visualised as green vectors. Atoms are represented as solid spheres: hydrogen (green), boron (pink), and oxygen (red). Mode indices correspond to those listed in Table S1. The inset in the left panel shows the equilibrium (undisplaced) molecular structure for reference.

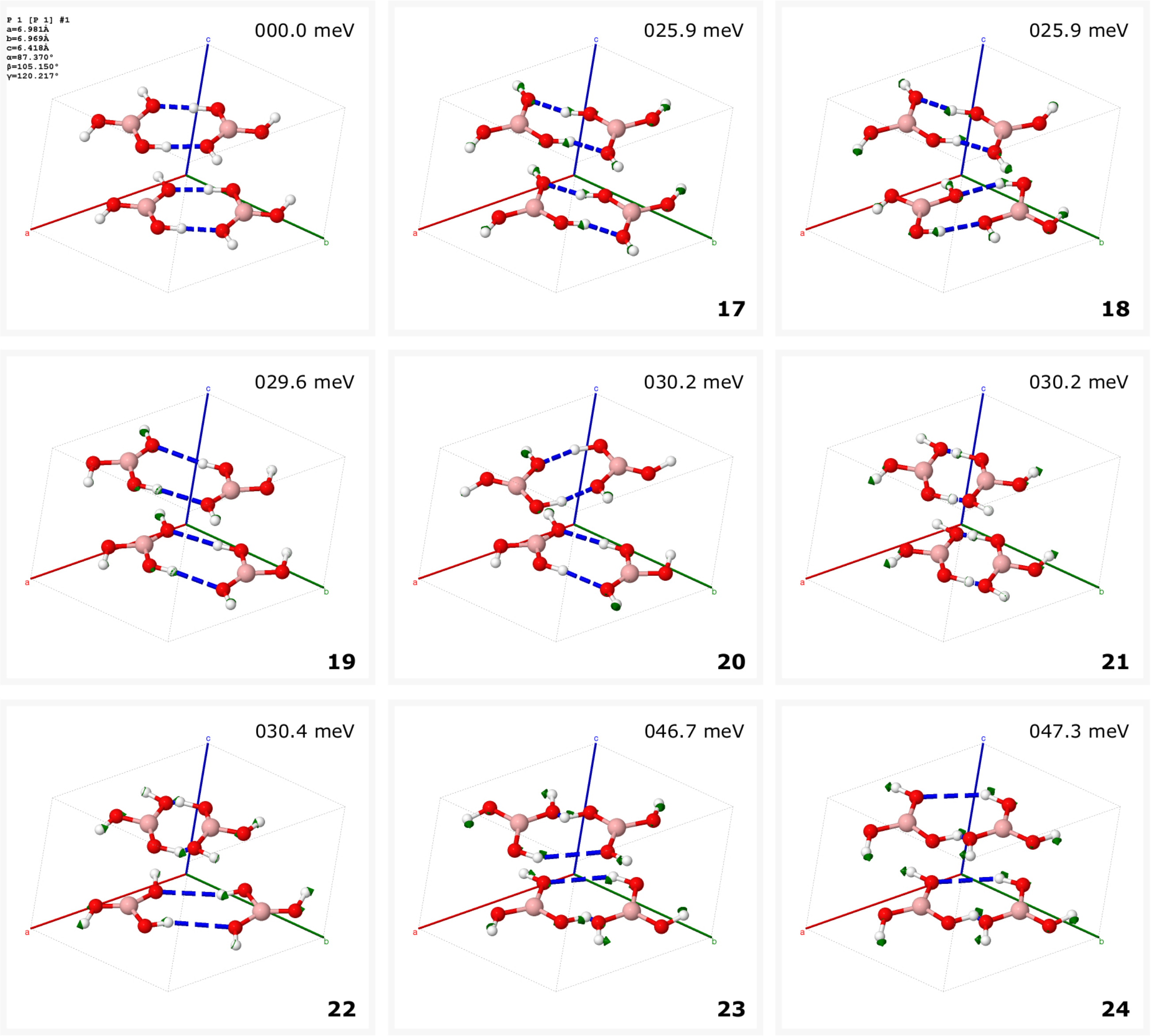

Figure S5. Zone-centre vibrational eigenmode displacement patterns for triclinic (*P*-1) boric acid (modes No. 17–24), computed via harmonic lattice dynamics (HLD) using DFPT and the PBE-TS functional. The atomic displacements are visualised as green vectors. Atoms are represented as solid spheres: hydrogen (green), boron (pink), and oxygen (red). Mode indices correspond to those listed in Table S1. The inset in the left panel shows the equilibrium (undisplaced) molecular structure for reference.

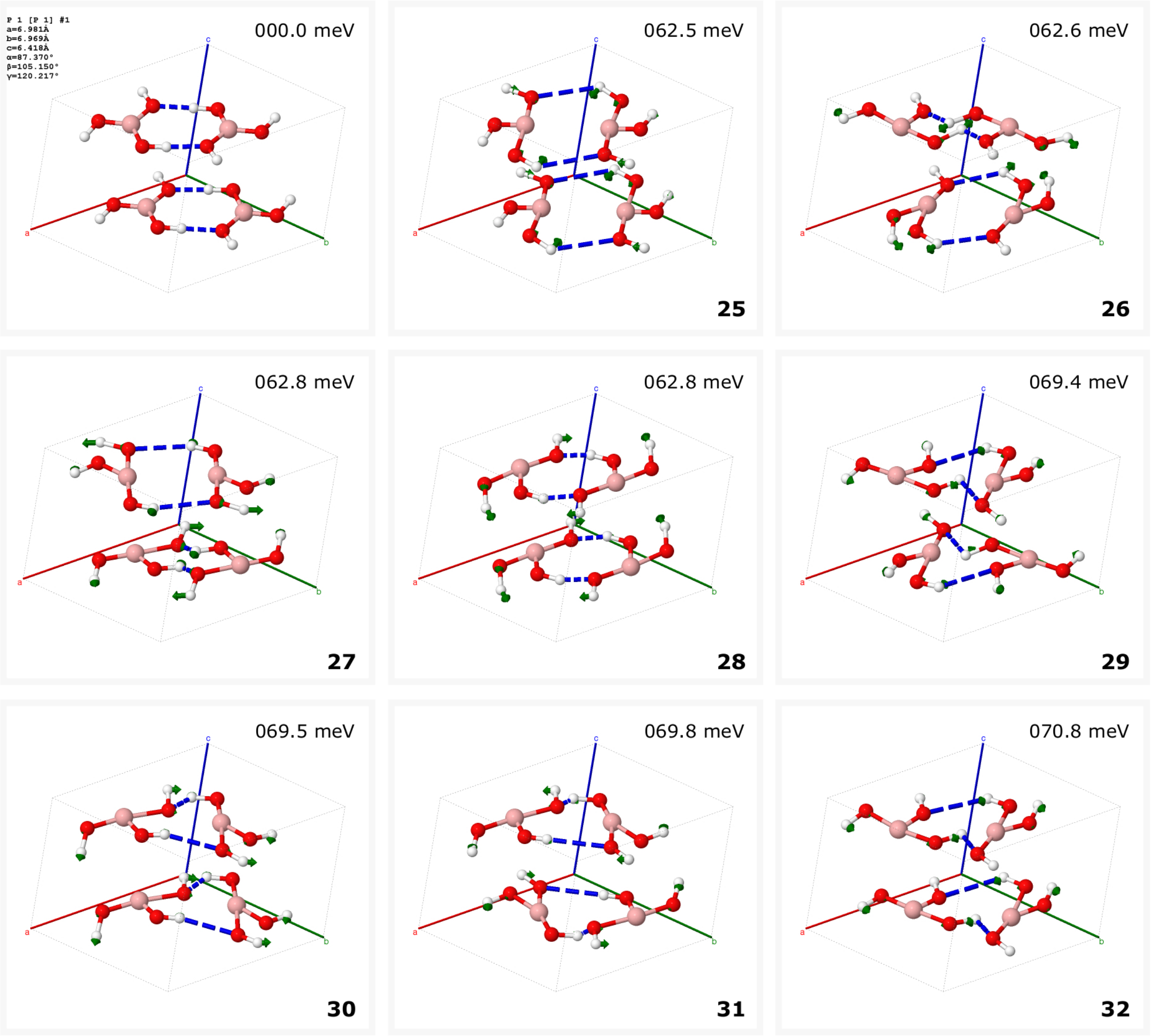

Figure S6. Zone-centre vibrational eigenmode displacement patterns for triclinic (*P*-1) boric acid (modes No. 25–32), computed via harmonic lattice dynamics (HLD) using DFPT and the PBE-TS functional. The atomic displacements are visualised as green vectors. Atoms are represented as solid spheres: hydrogen (green), boron (pink), and oxygen (red). Mode indices correspond to those listed in Table S1. The inset in the left panel shows the equilibrium (undisplaced) molecular structure for reference.

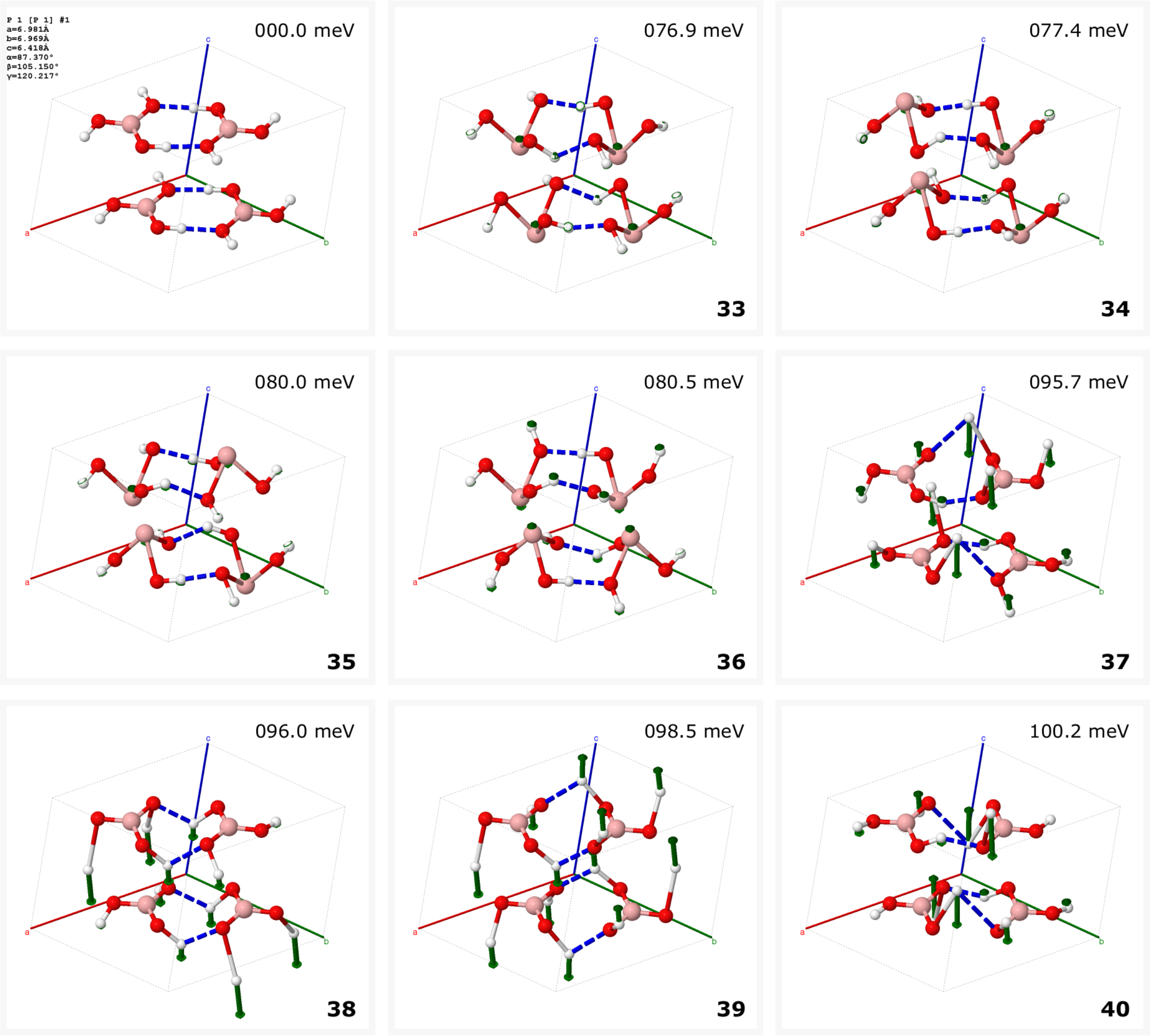

Figure S7. Zone-centre vibrational eigenmode displacement patterns for triclinic (*P*-1) boric acid (modes No. 33–40), computed via harmonic lattice dynamics (HLD) using DFPT and the PBE-TS functional. The atomic displacements are visualised as green vectors. Atoms are represented as solid spheres: hydrogen (green), boron (pink), and oxygen (red). Mode indices correspond to those listed in Table S1. The inset in the left panel shows the equilibrium (undisplaced) molecular structure for reference.

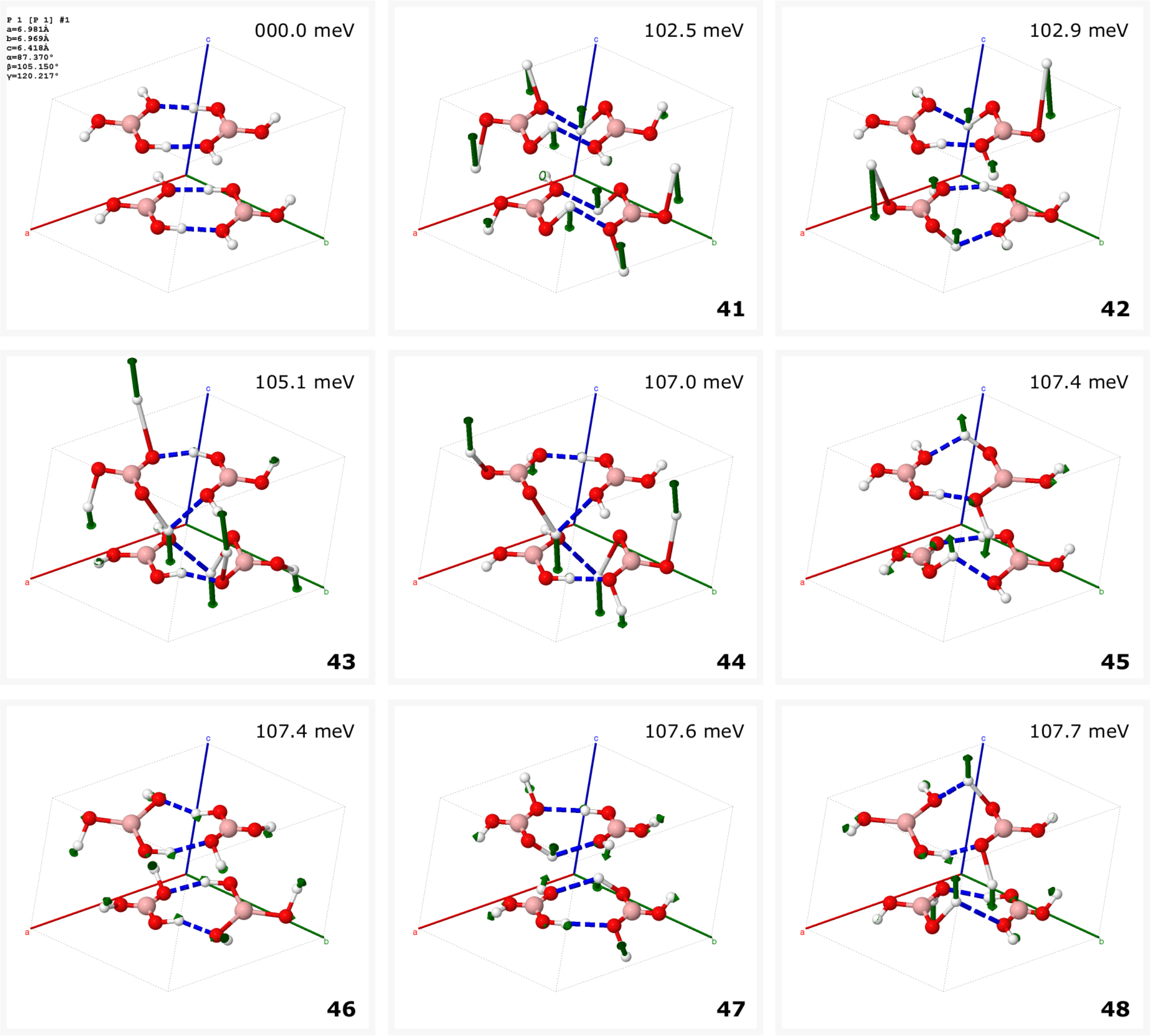

Figure S8. Zone-centre vibrational eigenmode displacement patterns for triclinic (*P*-1) boric acid (modes No. 41–48), computed via harmonic lattice dynamics (HLD) using DFPT and the PBE-TS functional. The atomic displacements are visualised as green vectors. Atoms are represented as solid spheres: hydrogen (green), boron (pink), and oxygen (red). Mode indices correspond to those listed in Table S1. The inset in the left panel shows the equilibrium (undisplaced) molecular structure for reference.

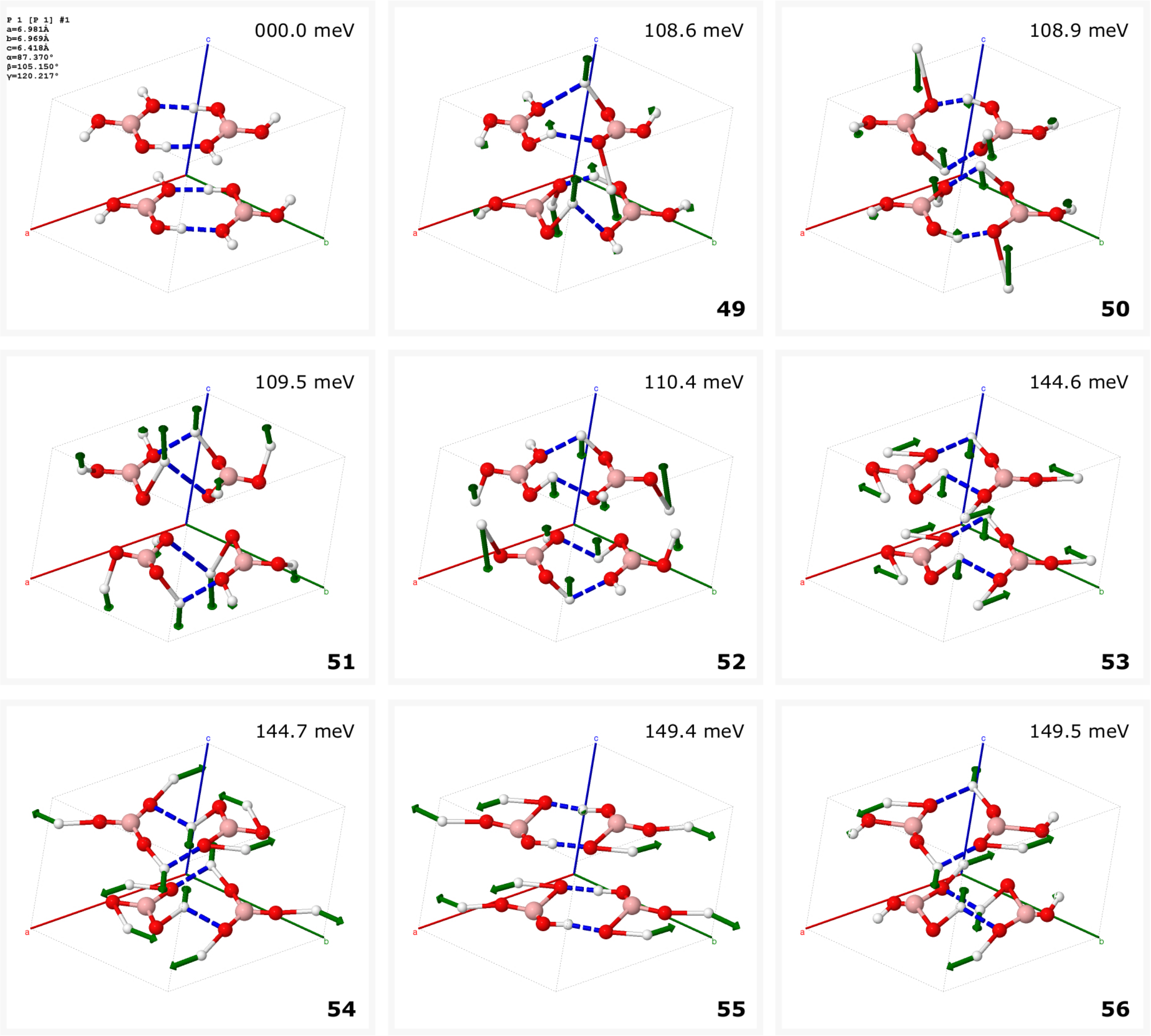

Figure S9. Zone-centre vibrational eigenmode displacement patterns for triclinic (*P*-1) boric acid (modes No. 49–56), computed via harmonic lattice dynamics (HLD) using DFPT and the PBE-TS functional. The atomic displacements are visualised as green vectors. Atoms are represented as solid spheres: hydrogen (green), boron (pink), and oxygen (red). Mode indices correspond to those listed in Table S1. The inset in the left panel shows the equilibrium (undisplaced) molecular structure for reference.

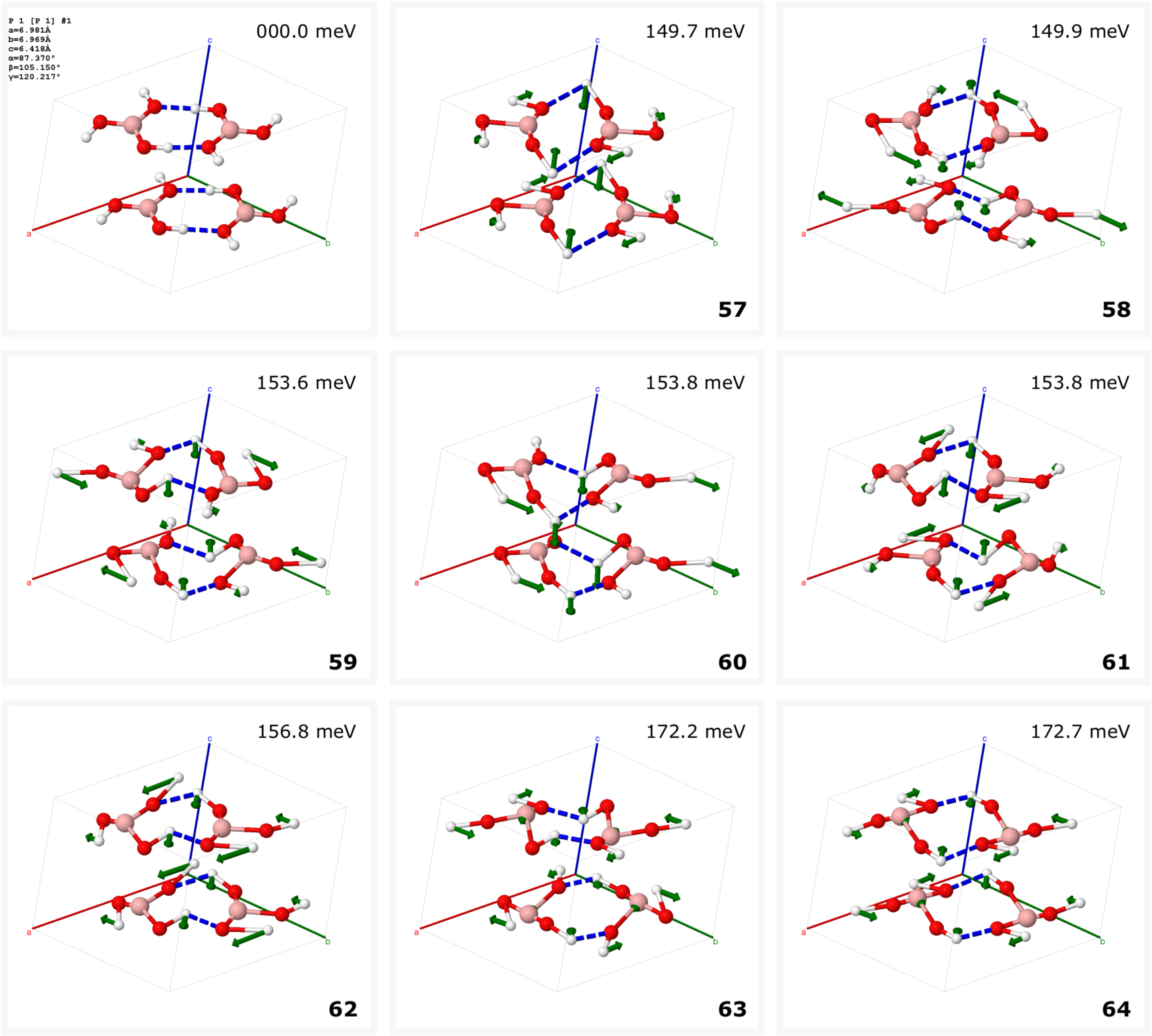

Figure S10. Zone-centre vibrational eigenmode displacement patterns for triclinic (*P*-1) boric acid (modes No. 57–64), computed via harmonic lattice dynamics (HLD) using DFPT and the PBE-TS functional. The atomic displacements are visualised as green vectors. Atoms are represented as solid spheres: hydrogen (green), boron (pink), and oxygen (red). Mode indices correspond to those listed in Table S1. The inset in the left panel shows the equilibrium (undisplaced) molecular structure for reference.

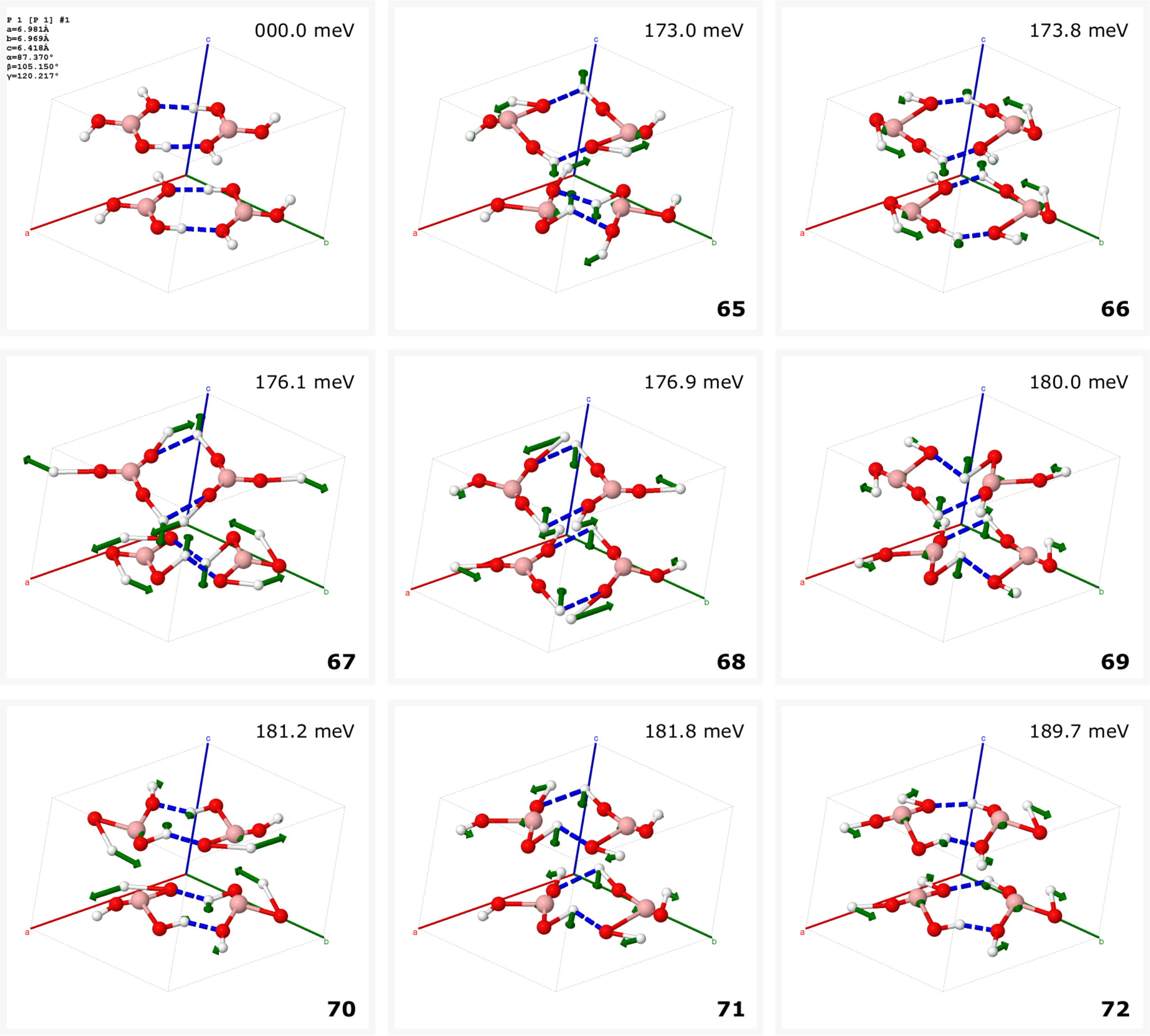

Figure S11. Zone-centre vibrational eigenmode displacement patterns for triclinic (*P*-1) boric acid (modes No. 65–72), computed via harmonic lattice dynamics (HLD) using DFPT and the PBE-TS functional. The atomic displacements are visualised as green vectors. Atoms are represented as solid spheres: hydrogen (green), boron (pink), and oxygen (red). Mode indices correspond to those listed in Table S1. The inset in the left panel shows the equilibrium (undisplaced) molecular structure for reference.

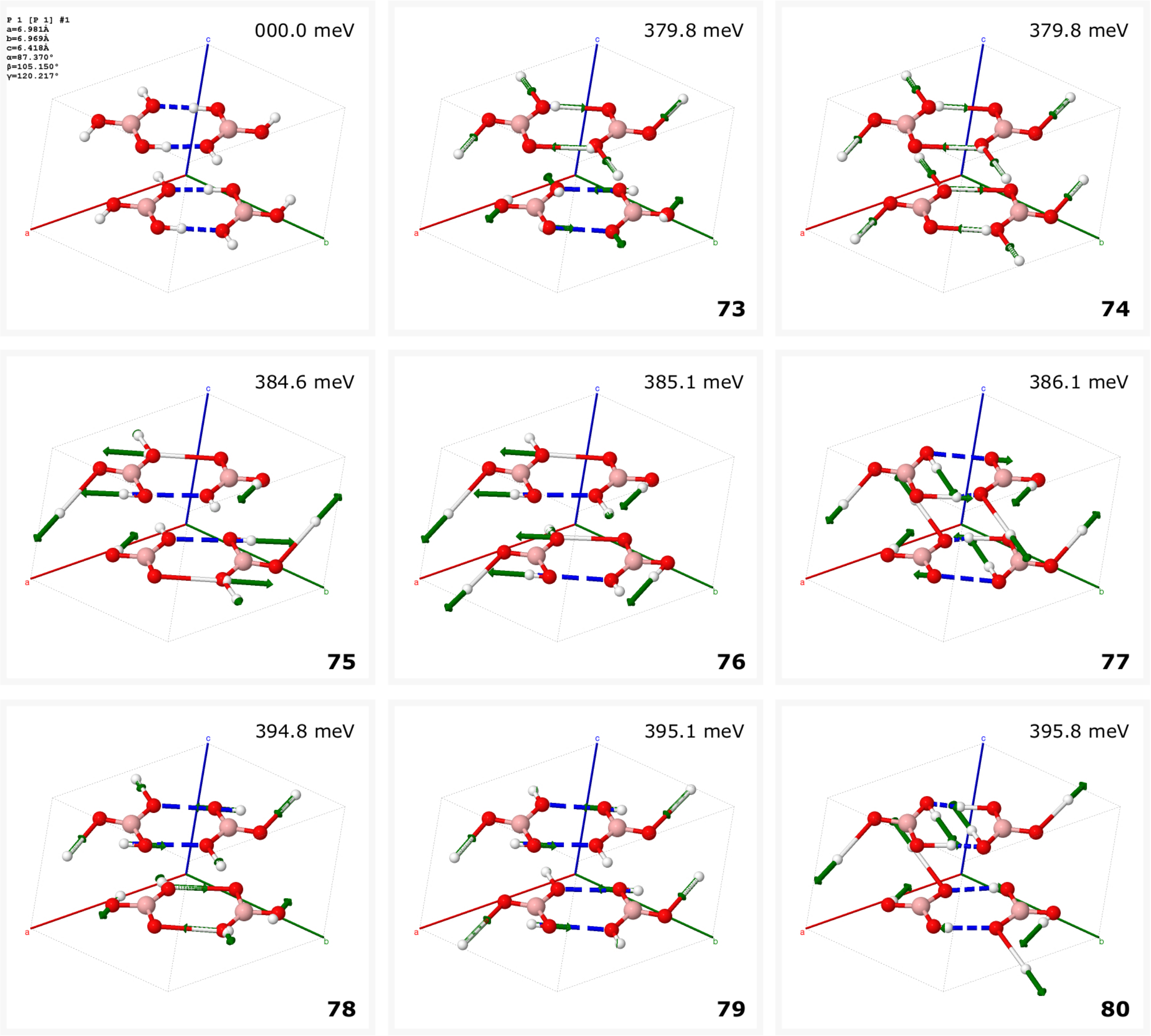

Figure S12. Zone-centre vibrational eigenmode displacement patterns for triclinic (P-1) boric acid (modes No. 73–80), computed via harmonic lattice dynamics (HLD) using DFPT and the PBE-TS functional. The atomic displacements are visualised as green vectors. Atoms are represented as solid spheres: hydrogen (green), boron (pink), and oxygen (red). Mode indices correspond to those listed in Table S1. The inset in the left panel shows the equilibrium (undisplaced) molecular structure for reference.

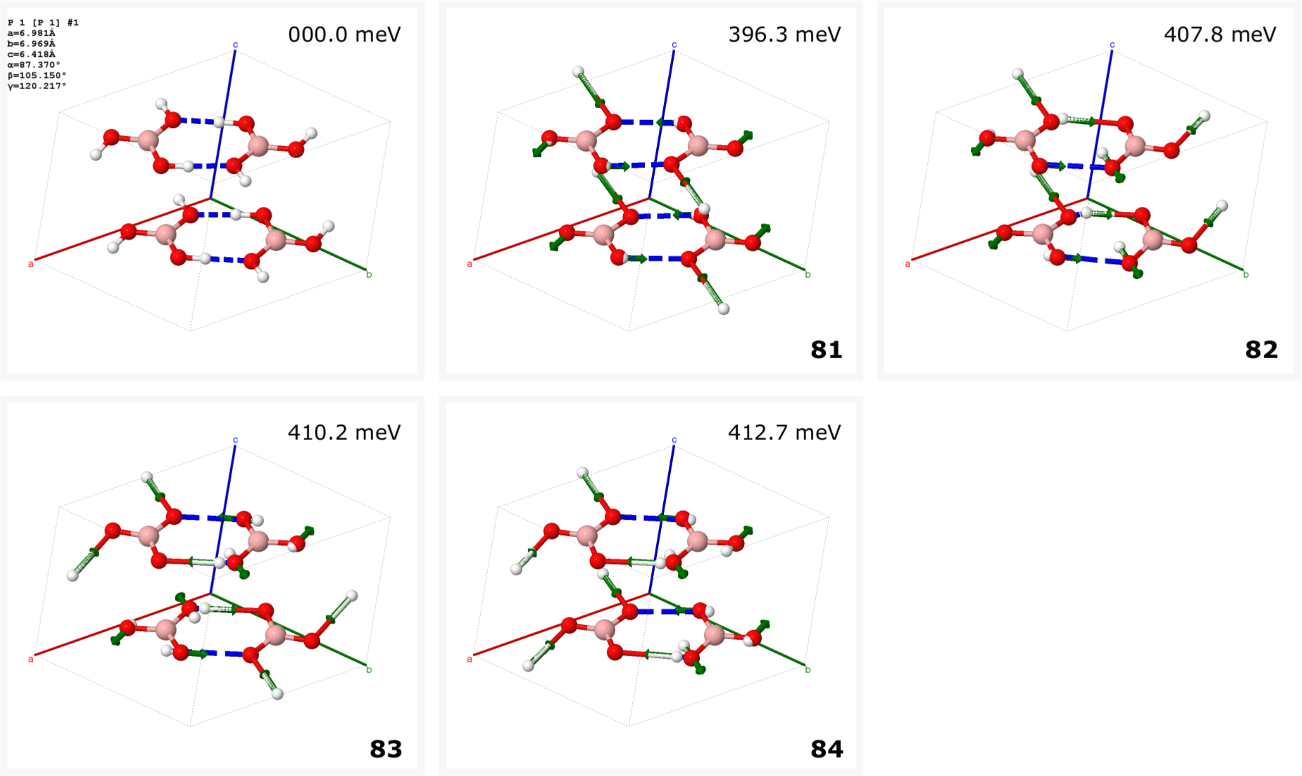

Figure S13. Zone-centre vibrational eigenmode displacement patterns for triclinic (*P*-1) boric acid (modes No. 81–84), computed via harmonic lattice dynamics (HLD) using DFPT and the PBE-TS functional. The atomic displacements are visualised as green vectors. Atoms are represented as solid spheres: hydrogen (green), boron (pink), and oxygen (red). Mode indices correspond to those listed in Table S1. The inset in the left panel shows the equilibrium (undisplaced) molecular structure for reference.

S3 Phonon properties beyond the Γ-point

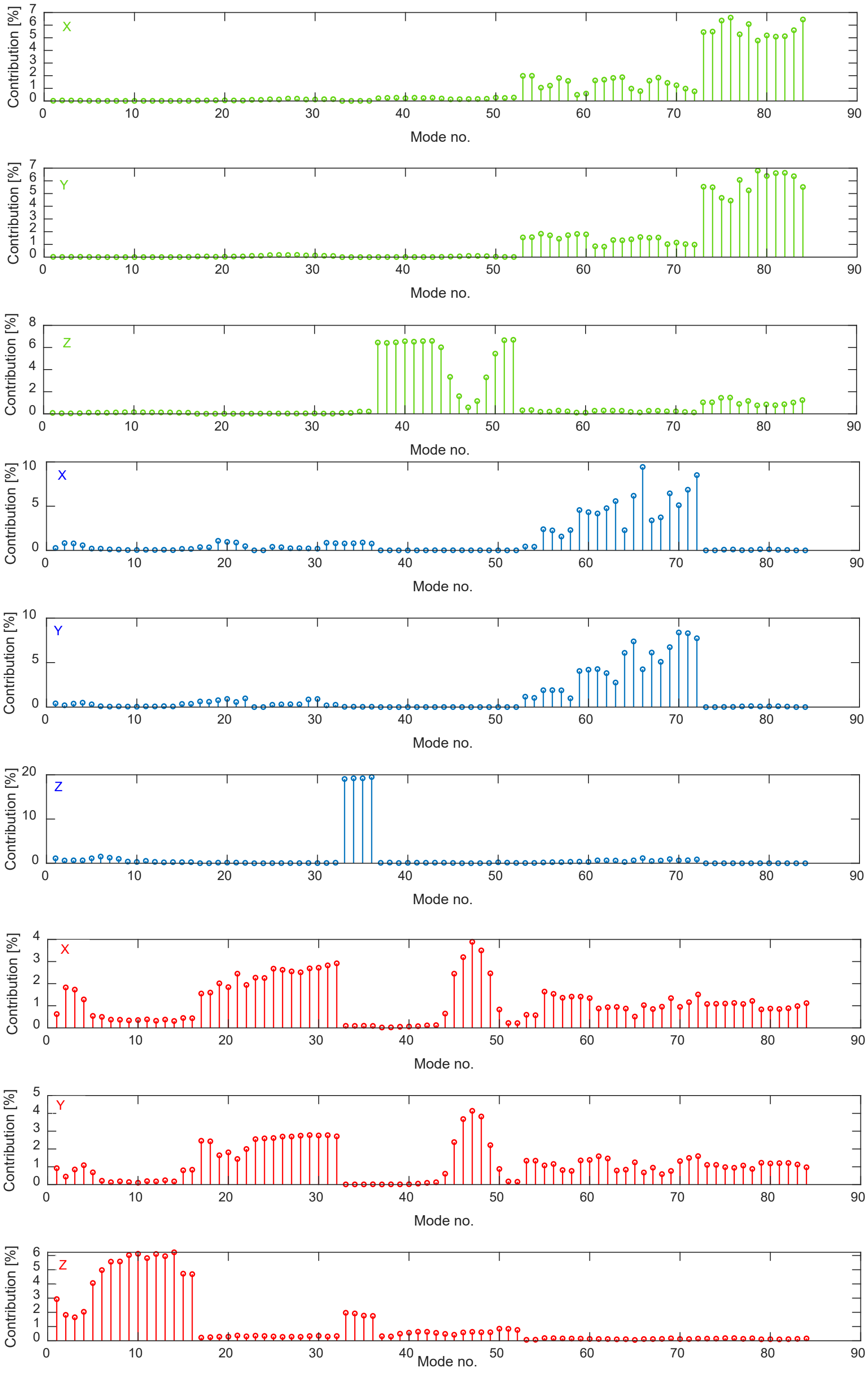

Figure S14. q-averaged individual mode contributions to the total kinetic energy of protons (top panel), boron (middle panel), and oxygen (bottom panel) in boric acid along the X, Y, and Z crystallographic axes. For details, see the text of the main article.

A Calculation of the isotope effect on the widths of the nuclear momentum distributions and the zero-point vibrational energies of boron

In neutron Compton scattering (NCS), it is customary to express momentum transfer q in Å^{-1}, energy transfer ω in meV, and nuclear mass M in atomic mass units (amu). With this choice of units, the reduced Planck constant has the numerical value $\hbar$ = 2.04458 (meV·amu)$^{1/2}$ Å. Consequently, the nuclear kinetic energy of a target nucleus of mass (M) can be written as:

$$E_{\text{recoil}} \frac{3\hbar^2\sigma^2}{2M} = \frac{3\times 2.04458^2}{2M}\sigma^2(\text{meV}) \tag{1}$$

where M is in amu and σ in Å^{-1}.

In the harmonic approximation, the ground-state momentum distribution of a single atom is Gaussian with

$$\sigma_p = \sqrt{\langle p^2\rangle} = \sqrt{\frac{m\hbar\omega}{2}}, \tag{2}$$

$$\omega = \sqrt{\frac{k}{m}}. \tag{3}$$

Since

$$\sigma_p = \sqrt{\frac{\hbar}{2}}(mk)^{1/4} \propto m^{1/4}, \tag{4}$$

it follows that for two isotopes i and j,

$$\frac{\sigma_i}{\sigma_j} = \left(\frac{m_i}{m_j}\right)^{1/4}. \tag{5}$$

Let f_{10}=0.199 and f_{11}=0.801 be the natural abundances of ^{10}B and ^{11}B. The measured width σ_{nat} =9.6 Å^{-1} for natural-abundance boron corresponds to a mixture of two Gaussians. Its second moment is

$$\sigma_{\text{nat}}^2 = f_{10}\,\sigma_{10}^2 + f_{11}\,\sigma_{11}^2. \tag{6}$$

Define

$$\alpha = \left(\tfrac{m_{10}}{m_{11}}\right)^{1/4} = \left(\tfrac{10}{11}\right)^{1/4} \approx 0.97646, \tag{7}$$

so that $\sigma_{10} = \alpha\,\sigma_{11}$. Then

$$\sigma_{\text{nat}}^2 = f_{10}\,(\alpha\,\sigma_{11})^2 + f_{11}\,\sigma_{11}^2 = \sigma_{11}^2\left(f_{10}\,\alpha^2 + f_{11}\right), \tag{8}$$

Hence σ_{11} = 9.64 Å^{-1} and σ_{10} =9.42 Å^{-1}.

The zero-point-energy isotope effect is $\Delta ZPE = ZPE_{10} - ZPE_{11} = 111.2 - 105.9$ = 5.3 meV/atom = 0.5 kJ/mol.

All results assume a common force constant k for both isotopes (i.e., the Born–Oppenheimer approximation).

B Isotope effects in the atom-projected VDoS spectra of boric acid

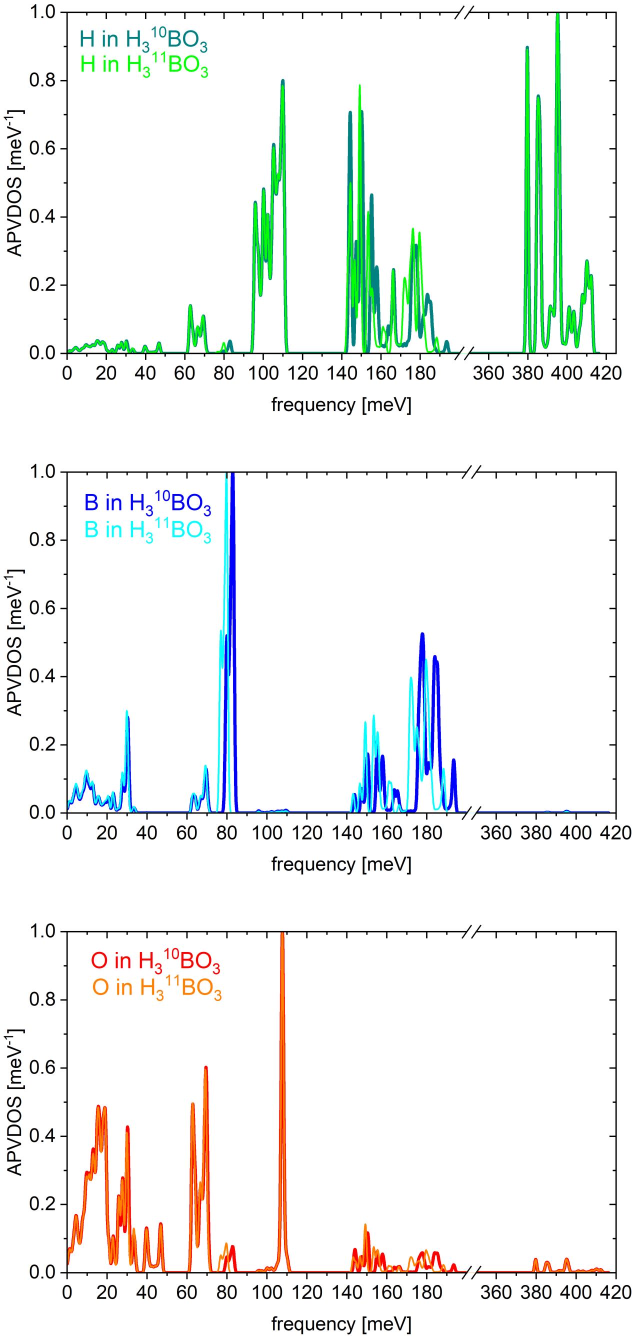

Figure S15. Isotope effects in the atom-projected VDoS spectra of the triclinic form (*P*-1) of the hydrogenous $H_3{}^{11}BO_3$ and $H_3{}^{10}BO_3$

C Isotope effects in the INS spectra of boric acid

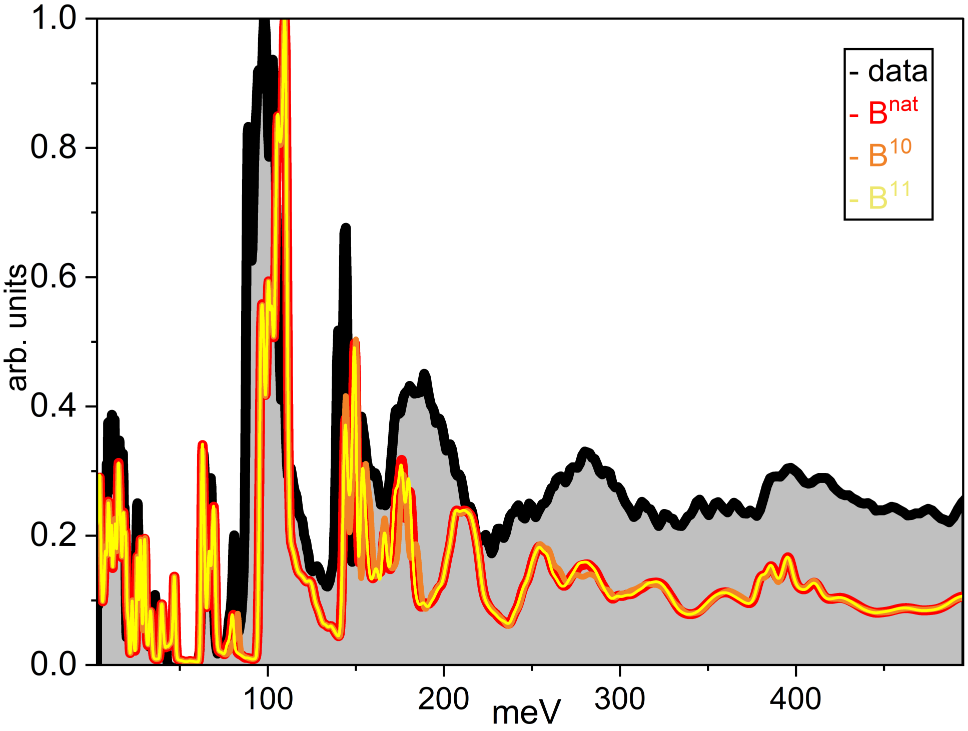

Figure S16. Comparison between the experimental INS spectrum of the triclinic form (*P*-1) of the hydrogenous $H_3{}^{11}BO_3$ by Parker[1] and the theoretical spectra according to low-temperature HLD (0 K) simulations of $H_3{}^{11}BO_3$, $H_3{}^{10}BO_3$, and $H_3{}^{nat}BO_3$

D Isotope effects in total cross-section of boric acid

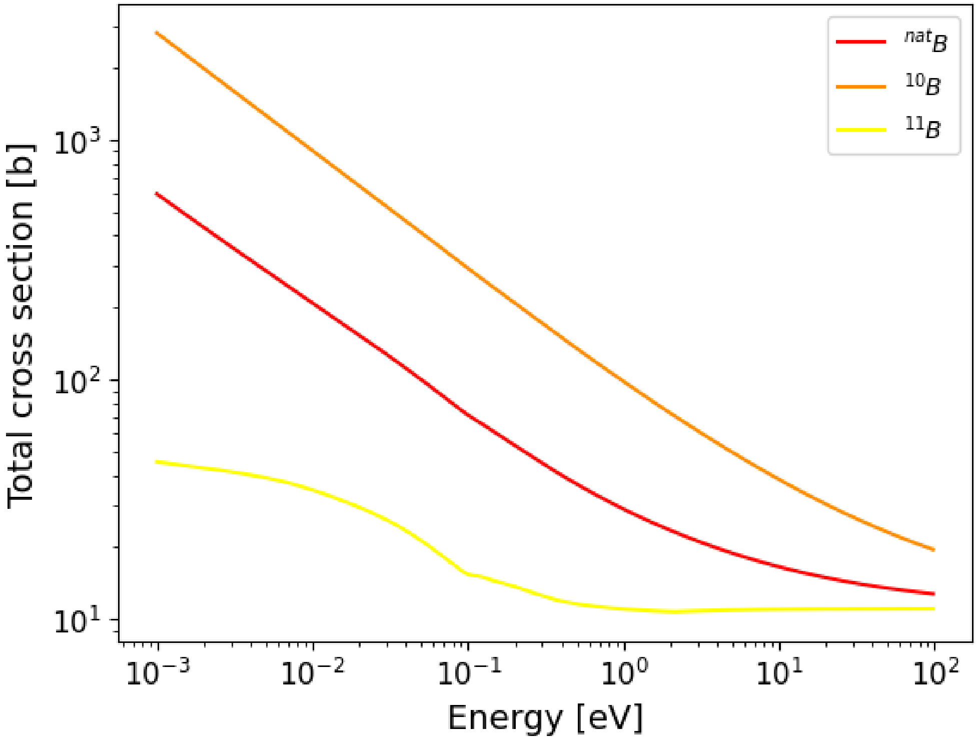

Figure S17. Room temperature simulations of the total cross-section curves as functions of the incident neutron energy of the triclinic form (*P*-1) of the hydrogenous boric acid. The simulations were performed in the NCRYSTAL environment based on the atom-projected vibrational densities of states of hydrogen, oxygen and ^{10}B, ^{11}B, and ^{nat}B boron obtained from the HLD (0 K) for $H_3{}^{11}BO_3$, $H_3{}^{10}BO_3$, and $H_3{}^{nat}BO_3$

References

1. Parker, S. F. Inelastic neutron scattering spectrum of boric acid, $B(OH)_3$. http://dx.doi.org/10.5286/edata/101 (2015). Measured on the TOSCA instrument with 99% ^{11}B enrichment.
